# The amyloid plaque proteome in early onset Alzheimer’s disease and Down syndrome

**DOI:** 10.1186/s40478-022-01356-1

**Published:** 2022-04-13

**Authors:** Eleanor Drummond, Tomas Kavanagh, Geoffrey Pires, Mitchell Marta-Ariza, Evgeny Kanshin, Shruti Nayak, Arline Faustin, Valentin Berdah, Beatrix Ueberheide, Thomas Wisniewski

**Affiliations:** 1 Brain and Mind Centre and School of Medical Sciences, Faculty of Medicine and Health, University of Sydney, 94 Mallett Street, Camperdown, NSW Australia; 2 Centre for Cognitive Neurology, Department of Neurology, New York University Grossman School of Medicine, Science Building, Rm 1017, 435 East 30th Street, New York, NY 10016 USA; 3 Proteomics Laboratory, Division of Advanced Research Technologies, New York University Grossman School of Medicine, New York, NY USA; 4 Present Address: Merck & Co., Inc, Computational & Structural Chemistry, Kenilworth, NJ USA; 5 Department of Biochemistry and Molecular Pharmacology, NYU Grossman School of Medicine, New York, NY USA; 6 Departments of Pathology and Psychiatry, Neuroscience Institute, New York University Grossman School of Medicine, New York, NY USA

**Keywords:** Alzheimer’s disease, Amyloid plaques, Amyloid beta, Proteomics, Early onset, Down syndrome, Mass spectrometry

## Abstract

Amyloid plaques contain many proteins in addition to beta amyloid (Aβ). Previous studies examining plaque-associated proteins have shown these additional proteins are important; they provide insight into the factors that drive amyloid plaque development and are potential biomarkers or therapeutic targets for Alzheimer’s disease (AD). The aim of this study was to comprehensively identify proteins that are enriched in amyloid plaques using unbiased proteomics in two subtypes of early onset AD: sporadic early onset AD (EOAD) and Down Syndrome (DS) with AD. We focused our study on early onset AD as the drivers of the more aggressive pathology development in these cases is unknown and it is unclear whether amyloid-plaque enriched proteins differ between subtypes of early onset AD. Amyloid plaques and neighbouring non-plaque tissue were microdissected from human brain sections using laser capture microdissection and label-free LC–MS was used to quantify the proteins present. 48 proteins were consistently enriched in amyloid plaques in EOAD and DS. Many of these proteins were more significantly enriched in amyloid plaques than Aβ. The most enriched proteins in amyloid plaques in both EOAD and DS were: COL25A1, SMOC1, MDK, NTN1, OLFML3 and HTRA1. Endosomal/lysosomal proteins were particularly highly enriched in amyloid plaques. Fluorescent immunohistochemistry was used to validate the enrichment of four proteins in amyloid plaques (moesin, ezrin, ARL8B and SMOC1) and to compare the amount of total Aβ, Aβ40, Aβ42, phosphorylated Aβ, pyroglutamate Aβ species and oligomeric species in EOAD and DS. These studies showed that phosphorylated Aβ, pyroglutamate Aβ species and SMOC1 were significantly higher in DS plaques, while oligomers were significantly higher in EOAD. Overall, we observed that amyloid plaques in EOAD and DS largely contained the same proteins, however the amount of enrichment of some proteins was different in EOAD and DS. Our study highlights the significant enrichment of many proteins in amyloid plaques, many of which may be potential therapeutic targets and/or biomarkers for AD.

**Supplementary Information:**

The online version contains supplementary material available at 10.1186/s40478-022-01356-1.

## Introduction

Amyloid plaques are a neuropathological hallmark of Alzheimer’s disease and primarily consist of the protein beta amyloid (Aβ). However, it is often overlooked that amyloid plaques also contain hundreds of proteins in addition to Aβ. These include proteins that directly interact with Aβ (e.g. apolipoprotein E [1]), proteins present in microglia and astrocytes that surround and infiltrate plaques, and proteins present in dystrophic neurites (e.g. phosphorylated tau [2], neurofilament proteins [3], secernin-1 [4]). Previous studies have shown that many of these plaque proteins have mechanistic roles in AD. For example, proteins that directly interact with Aβ influence Aβ aggregation and therefore mediate amyloid plaque formation [5–7]. The proteins present in plaque-associated glia influence glial function and can mediate pathological glial function [8, 9]. Proteins present in dystrophic neurites provide insight into the factors involved in the formation of dystrophic neurites and neuritic plaques, which correlate better with cognitive impairment than diffuse plaques [10]. Therefore, comprehensively profiling the proteins that are enriched in amyloid plaques would increase our understanding about AD pathogenesis, and possibly identify new biomarkers and/or new therapeutic targets for AD.

Previous studies have typically used immunohistochemistry to identify amyloid plaque proteins. Mass spectrometry-based proteomics is an alternative approach that allows efficient quantification of thousands of amyloid plaque proteins simultaneously. Proteomics also offers additional advantages of allowing discovery of novel plaque proteins due to its unbiased nature and bypassing complications due to antibody sensitivity and specificity issues. Given these significant advantages, we recently developed a localized proteomics approach to analyze the proteome of neuropathological lesions in AD such as plaques and neurofibrillary tangles [11–13].

The significant heterogeneity in the clinical and neuropathological phenotype of AD suggests that multiple subtypes of AD exist. Previous studies have used various approaches to define AD subtypes [14–17]. Some studies have defined AD subtypes by age of onset (e.g. early onset vs late onset), genetics (e.g. apoE2 vs apoE3 vs apoE4 or familial AD vs sporadic AD), by neuropathology phenotype (e.g. limbic predominant vs hippocampal sparing vs typical), by rate of progression (e.g. rapidly progressive AD vs typical AD), or more recently using unbiased ‘omics approaches. We recently showed that plaques in rapidly progressive AD had a significantly different proteome than plaques in typical sporadic AD, suggesting that the amyloid plaque proteome is not consistent in all AD subtypes and that these plaque protein differences may contribute to the development of different subtypes of AD [11]. It is currently unclear whether amyloid plaques in other AD subtypes also have significantly different protein composition, or whether these plaque protein differences were unique to rapidly progressive AD.

The aim of this study was to compare the amyloid plaque proteome in two subtypes of early onset AD: sporadic early onset AD (EOAD) and Down Syndrome (DS) with AD. Between 5 and 10% of AD cases are considered early onset [18]. Of these, only approximately 10% are caused by *APP*, *PSEN1* and *PSEN2* mutations. The cause of the remaining ~ 90% of EOAD cases is unknown and these cases are therefore characterized as sporadic EOAD. It is currently unclear if the same molecular mechanisms drive sporadic EOAD cases and late-onset AD [18]. DS with AD is another prevalent subtype of early onset AD. Adults with DS have a very high risk of developing AD, which is thought to be driven by the triplication and consequent overexpression of APP in DS [19]. People with DS develop AD associated neuropathology very early in life. Accumulation of soluble Aβ has been observed in fetuses with DS [20]. Intraneuronal Aβ is present in children as young as 1 year old [21], which is followed by the development of diffuse plaques by the age of approximately 12 years [22, 23]. Mature plaques are commonly present in the 30’s and advanced AD neuropathology is present by the 40’s [24]. The progressive accumulation of amyloid and tau pathology in DS largely follows a similar pattern to that observed in AD [25], albeit with more plaques in the striatum and thalamus [26] and a higher plaque density overall in DS in comparison to AD [27]. Multiple studies have shown that plaques in DS contain similar post-translationally modified Aβ species as observed in AD, including Aβ phosphorylated at serine 8 and pyroglutamate modified Aβ [23, 28–31], however it is still unknown if plaques in DS have a different protein composition to that in AD.

Here, we show that amyloid plaques in DS and EOAD are enriched in many proteins besides Aβ including a common core group of 48 proteins that are enriched in plaques in both AD subtypes. While similar proteins were enriched in both DS and EOAD, some proteins were enriched to a greater extent in plaques in a particular subtype of AD, providing new evidence that some distinctions in plaque protein composition are present.

## Methods

### Ethics statement

All procedures were performed under protocols approved by the Institutional Review Board at New York University Alzheimer Disease Center, NY, USA. In all cases, written informed consent for research was obtained from the patient or legal guardian, and the material used had appropriate ethical approval for use in this project. All patients’ data and samples were coded and handled according to NIH guidelines to protect patients’ identities.

### Human tissue samples

N = 5 cases of early onset sporadic Alzheimer’s disease (EOAD) and n = 5 cases of DS with Alzheimer’s disease were included for proteomic experiments. Inclusion criteria for EOAD included age < 65 years, ABC neuropathological score of A3, B3, C3 [32], no mutation in *APP*, *PSEN1* or *PSEN2*, tissue formalin fixation time < 6 months. Inclusion criteria for DS cases was ABC neuropathological score of A3, B3, C3, formalin fixation time < 6 months. Formalin fixed paraffin embedded tissue blocks containing the hippocampus and surrounding entorhinal/temporal cortex that were collected and processed as part of routine autopsy procedures were used in this study. This region was selected because it contains a high amount of amyloid pathology in EOAD and in DS with AD. N = 3 cases of EOAD, DS, late onset sporadic AD (LOAD) and cognitively normal, age matched controls were included in immunohistochemistry validation studies. Case specific information for the human tissue samples used in this study is included in Table 1.

**Table 1:** Human tissue samples used in this study

Patient ID	Sex	Age at death	*APOE* genotype on FFPE or FT	ABC score	Fixation duration in weeks	Inclusion in proteomics study	Inclusion in IHC studies	Number of plaques micro-dissected	Number of non-plaques micro-dissected
EOAD #1	F	55	*ɛ*3/*ɛ*3; FFPE	A3, B3, C3	2	Yes	Yes	641	643
EOAD #2	M	62	*ɛ*3/*ɛ*3; FT	A3, B3, C3	3	Yes	Yes	622	622
EOAD #3	M	63	*ɛ*3/*ɛ*3; FFPE	A3, B3, C3	2	Yes	Yes	644	648
EOAD #4	M	63	*ɛ*4/*ɛ*4; FT	A3, B3, C3	3	Yes	Yes	627	627
EOAD #5	F	60	*ɛ*3/*ɛ*4; FT	A3, B3, C3	3	Yes	Yes	680	680
EOAD #6	M	70	*ɛ*3/*ɛ*3; FFPE	A3, B3, C3	2		Yes	n/a	n/a
EOAD #7	F	70	*ɛ*3/*ɛ*3; FFPE	A3, B3, C3	2		Yes	n/a	n/a
DS #1	F	58	*ɛ*3/*ɛ*3; FFPE	A3, B3, C3	2	Yes	Yes	607	607
DS #2	M	55	*ɛ*3/*ɛ*4; FFPE	A3, B3, C3	2	Yes	Yes	641	641
DS #3	M	54	*ɛ*3/*ɛ*3; FFPE	A3, B3, C3	2	Yes	Yes	603	603
DS #4	F	59	*ɛ*3/*ɛ*3; FFPE	A3, B3, C3	2	Yes	Yes	633	633
DS #5	F	37	*ɛ*3/*ɛ*3; FFPE	A3, B3, C3	2	Yes	Yes	626	624
LOAD #1	M	76	ɛ3/ɛ4; FT	A3, B3, C3	3		Yes	n/a	n/a
LOAD #2	M	77	ɛ3/ɛ3; FFPE	A3, B3, C3	3		Yes	n/a	n/a
LOAD #3	F	88	ɛ3/ɛ4; FT	A3, B3, C3	2		Yes	n/a	n/a
Control #1	M	59	*ɛ*3/*ɛ*3; FFPE	A1, B1, C0	3		Yes	n/a	n/a
Control #2	F	77	*ɛ*3/*ɛ*3; FFPE	A1, B1, C1	2		Yes	n/a	n/a
Control #3	F	71	*ɛ*3/*ɛ*3; FFPE	A1, B1, C0	2		Yes	n/a	n/a

### APOE genotyping

APOE genotyping was performed on all the cases using either formalin-fixed paraffin embedded (FFPE) or frozen tissue (FT) for the cases where it was available (see Table 1). FT is the preferred tissue for genotyping as the results are more reliable using this source, which is less likely to be affected by DNA contamination; however, FT was available only from five cases. For FFPE tissues, DNA was isolated from six 8 µm brain sections per sample, using the automated system QIAsymphony SP (Qiagen) and the protocol indicated by the manufacturer. Two endpoint PCRs were performed before sequencing. The first endpoint PCR was conducted in a total volume of 25 µl containing 0.2 µM of each custom primer (Forward primer 5′ AGGCCTACAAATCGGAACTGG 3′; reverse primer 5′ CCTGTTCCACCAGGGGC 3′; Sigma), 0.5 mM each dNTP (Thermo Scientific), 2 U GoTaq G2 Hot Start polymerase (Promega), 25 mM MgCl_2_ solution (Promega) and 4.2 µl Betaine (Sigma). Cycling conditions were at 98 °C for 4 min and 40 cycles at 98 °C/10 s, 63 °C/1 min and 72 °C/1 min 10 s, followed by 72 °C 10 min. All the amplified fragments were resolved on 2% agarose gels, stained with GelRed 10,000X (Biotium) and visualized under UV exposure. DNA was purified from the agarose gel using the Illustra™ GFX™ PCR DNA and Gel Band Purification Kit (Cytiva) as indicated by the manufacturer, and DNA concentration was quantified using nanodrop One (Thermo Scientific). The second endpoint PCR was performed using the purified DNA with the conditions described previously, except for the concentration of the primers, which was reduced to 0.15 µM. Unpurified PCR products were submitted to Genewiz for Sanger sequencing, and the sequences were analyzed using SnapGene 5.3.1 software (Additional File 2). For genotyping using frozen tissue, 25 mg were dissected from the brain section and transferred to a 1.5 ml tube. DNA was isolated using the DNeasy Blood & Tissue kit (Qiagen) following the manufacturer’s instructions. A single endpoint PCR was performed in a total volume of 25 µl containing 0.2 µM of each custom primer (Forward primer 5′ AGCCCTTCTCCCCGCCTCCCACTGT 3′; reverse primer 5′ CTCCGCCACCTGCTCCTTCACCTCG 3′; Sigma), 10 µl of DreamTaq Green PCR Master Mix (2X) and 4.2 µl Betaine (Sigma). Cycling conditions were at 98 °C for 4 min and 35 cycles at 98 °C/10 s, 63 °C/45 s and 72 °C/1 min 10 s, followed by 72 °C 10 min. Unpurified PCR products were submitted to Genewiz for Sanger sequencing, and the sequences were analyzed using SnapGene 5.3.1 software.

### Immunohistochemistry for Aβ species

8 µm formalin-fixed paraffin embedded tissue sections containing the hippocampus and surrounding cortex underwent fluorescent immunohistochemistry for six different Aβ species: total Aβ (combination of 4G8 [BioLegend; #800701] and 6E10 [BioLegend; #803001]), Aβ40 (in-house developed monoclonal rabbit antibody [33]), Aβ42 (in-house developed monoclonal rabbit antibody [33]), Aβ phosphorylated at serine position 8 (pAβ; in-house developed monoclonal mouse antibody), pyroglutamate modified Aβ (pyro-Aβ; [34]), and the conformational oligomeric antibody TWF9 [35] that recognizes beta-sheet containing oligomeric species including Aβ. Sections were deparaffinized and rehydrated through a series of xylene and ethanol washes. Antigen retrieval was performed by treatment with either 88% formic acid for 7 min followed by boiling in citrate buffer (10 mM sodium citrate, 0.05% Tween-20; pH6) for total Aβ, Aβ42, Aβ40, pyro-Aβ or with citrate buffer alone for pAβ and TWF9. Sections were blocked with 10% normal goat serum, incubated overnight primary antibodies diluted in 4% normal goat serum. Sections were incubated for 2 h at room temperature with appropriate fluorescent secondary antibodies (diluted 1:500, from Jackson ImmunoResearch). Sections were counter stained with Hoechst 33342 (Sigma) and coverslipped (Prolong Diamond, Thermo Fisher Scientific). Whole slide images were generated using a NanoZoomer HT2 (Hamamatsu) slide scanner. Eight 4× magnification images were collected for quantification from the whole slide scans: four containing the cortex, one each of CA1, CA2, CA3 and CA4, which together generated an average percentage staining load per case. Quantification of the percentage staining load was performed using ImageJ by quantifying the number of pixels above a defined staining threshold for each marker. The percentage staining load of total Aβ, Aβ42, Aβ40, phosphorylated Aβ and pyroglutamate Aβ abundance was restricted to staining in amyloid plaques only, while percentage staining load of oligomers refers to levels throughout the cortical grey matter. Significant differences were determined by one-way ANOVA followed by Tukey’s multiple comparisons test.

### Laser capture microdissection for localized proteomics

**Fig. 1:**
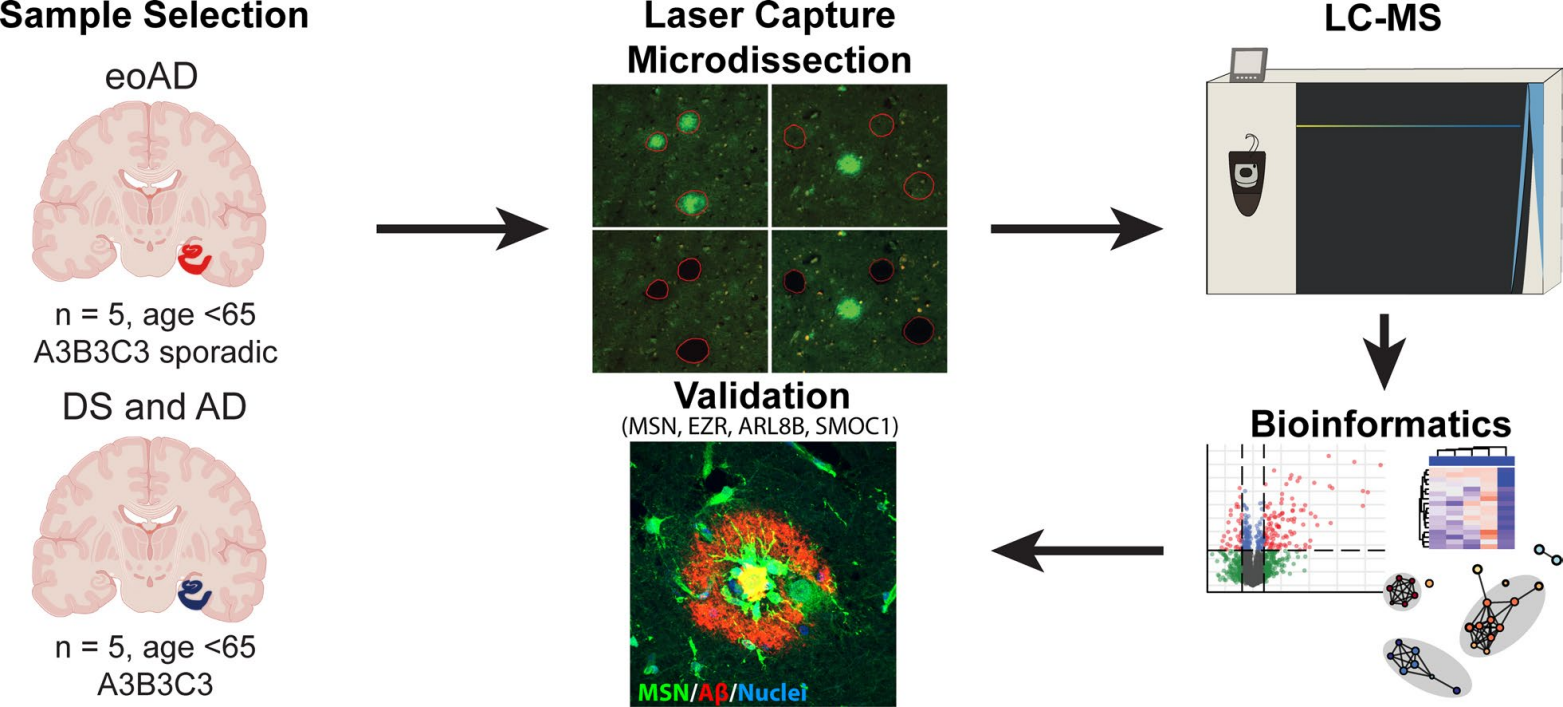
Schematic of methods used in this study. Formalin fixed paraffin embedded human tissue samples containing the hippocampus were used in this study (n = 5/group; all with advanced AD neuropathology [A3, B3, C3]). Laser capture microdissection was used to microdissect plaques or neighboring non-plaque tissue, LC–MS was used to quantify proteins present in each sample and various bioinformatics approaches were used to identify plaque enriched proteins and pathway or cell-type enrichment. Immunohistochemistry and comparison with previous studies through systematic literature searches was used to validate the enrichment of selected proteins in plaques

Proteomic studies were carried out using the method outlined in Fig. 1. 8 µm sections of formalin-fixed paraffin embedded tissue were collected onto laser capture microdissection (LCM)-compatible slides and amyloid plaques were visualized using fluorescent immunohistochemistry using a combination of the pan-Aβ antibodies 4G8 (1:4000; BioLegend; #800701) and 6E10 (1:4000; BioLegend; #803001). LCM was performed using a LMD 6500 microscope (Leica) using the method detailed in [11, 36]. 2 mm^2^ total area of fluorescently-labelled plaques was microdissected using LCM for each case. 2 mm^2^ total area of neighboring non-plaque tissue was also collected for each case. Non-plaque tissue was only selected from the same microscopic field of views that contained microdissected plaques, while remaining sufficiently distant from plaques to ensure that plaque-associated tissue was not collected (Fig. 1). The same number of microdissected regions were collected for plaques and non-plaques for each sample to control for proteomic variation based on the tissue loss associated with microdissection. The inclusion criteria for plaques in this study was any plaque visualized by IHC. There was no restriction based on plaque morphology. Plaques were microdissected from any region present on the hippocampal section, which included hippocampus, entorhinal cortex and temporal cortex. Plaques or non-plaque regions were collected into double distilled water and stored at − 80 °C until sample processing for LC–MS.

### Localized proteomics of amyloid plaques

Samples were processed for LC–MS/MS using the formic acid sample preparation method we have previously used to analyze the proteome of amyloid plaques [11, 13, 37]. Tissue underwent secondary deparaffinization using a heating protocol (95 °C for one hour and 65 °C for 2 h and were incubated in 70% LC–MS grade formic acid overnight at room temperature. Samples were sonicated (3 × 3 min), dried using a SpeedVac concentrator, resuspended in 100 mM ammonium bicarbonate and then reduced with Dithiothreitol (20 mM) and alkylated with iodoacetamide (50 mM). Samples were digested with sequencing grade modified trypsin (200 ng; Promega) by gentle agitation overnight at room temperature. Samples were acidified with 0.2% TFA and peptides were desalted using Poros beads. Peptides were eluted off the beads by addition of 40% acetonitrile in 0.5% acetic acid followed by the addition of 80% acetonitrile in 0.5% acetic acid. The organic solvent was removed using a SpeedVac concentrator and the samples were reconstituted in 0.5% acetic acid.

One third of each sample was loaded onto the column using the auto sampler of an EASY-nLC 1200 HPLC (ThermoFisher). The peptides were gradient eluted directly into an Orbitrap Fusion Lumos mass spectrometer using a 145-min gradient. The Orbitrap Fusion Lumos mass spectrometer acquired high resolution full MS spectra with a resolution of 240,000 (at m/z 200), AGC target of 1e6, with a maximum ion time of 50 ms, and scan range of 400–1500 m/z. Following each full MS data-dependent low resolution HCD MS/MS spectra were acquired. All MS/MS spectra were collected using the following instrument parameters: rapid ion trap scan rate, ACG target of 2e4, maximum ion time of 150 ms, one microscan, 0.7 m/z isolation window, fixed first mass of 150 m/z and NCE of 32.

### LC–MS data analysis

Protein quantitation was performed using MaxQuant software suite v. 1.6.3.4 [38]. Raw data generated by match between runs. The MS/MS spectra were searched against the SwissProt subset of the Uniprot *H. Sapiens* proteome database (26,186 entries) using the Andromeda search engine [39]. A list of 248 common laboratory contaminants included in MaxQuant, as well as reversed versions of all sequences were also added to the database. The enzyme specificity was set to trypsin with a maximum number of missed cleavages set to 2. Peptide identification was performed with an initial precursor mass deviation up to 7 ppm and a fragment mass deviation of 20 ppm with subsequent nonlinear mass recalibration. Oxidation of methionine and acetylation of protein NTerm were searched as variable modifications and carbamidomethylation of cysteines was searched as a fixed modification. The false discovery rate (FDR) for peptide, protein, and site identification was set to 1% and was calculated using a decoy database approach. The minimum peptide length was set to 7. The option match between runs (1 min time tolerance) was enabled to correlate identification and quantitation results across different runs. Normalization for label-free quantification was performed using MaxLFQ algorithm [38]. Missing values were imputed from normal distribution in Perseus [40] using default parameters. The final protein list was filtered to only include proteins that were present in at least 3 cases in at least one experimental group. An independent quantification for Aβ was manually curated and included in the search results, consistent with previous studies [41]. To do this, the intensity for Aβ was determined by integrating the area under the curve for peptide LVFFAEDVGSNK, which corresponds to amino acids 17–28 of Aβ.

Plaque enriched/depleted proteins were determined as those with a fold change difference between plaques and non-plaques > 1.5 fold and an uncorrected *p* value of *p* < 0.05 (paired *t*-test). Fold change difference was selected as the primary determinant of enrichment/depletion in plaques as this correlated best with immunohistochemistry studies, which is the gold standard approach for identifying plaque enriched proteins. Uncorrected *p*-values were included to provide an indication of variance within a group, however plaque-enriched proteins identified by *p*-values alone did not correlate as well with prior gold-standard immunohistochemistry studies.

Direct comparison of plaque protein levels in DS and EOAD was performed using plaque protein levels that were normalized to the neighboring non-plaque tissue for each individual case. For this, normalized plaque protein levels were calculated as the ratio of protein intensity in plaques:non-plaques for each case. Differences in normalized plaque protein levels between DS and EOAD were identified using an unpaired *t*-test and proteins were deemed significantly different based on a combination of *p* < 0.05 and fold change difference > 1.5.

### Data analysis and figure generation

General data manipulations and grouping were performed in R v4.0.2 [42] using the tidyverse v1.3.0 collection of packages. Plots were generated in R with the packages ggplot2 v3.3.2, ggpubr v0.4.0, ggrepel v0.8.2, EnhancedVolcano v1.6.0, VennDiagram v1.6.20, ComplexHeatmap v2.4.3, circlize v0.4.10 and edited in Adobe Illustrator v25.2.3. KEGG pathways and Gene Ontology enrichment analysis was performed in R using the packages enrichplot v1.8.1, DOSE v3.14.0, clusterProfiler 3.16.1, GOSemSim v2.14.2 with terms filtered to an FDR < 0.05. Heatmaps were created with scaled data using the scale function in R. Protein–protein interaction networks and gene ontology cellular compartment annotations were generated in STRING v11.0 [43] and the networks were edited in Cytoscape v3.8.1 and Adobe Illustrator.

### Comparison with previous studies

Systematic literature searches were used to identify plaque enriched proteins that have been validated in previous studies. A protein was designated a “known plaque protein” if there was published evidence of enrichment in amyloid plaques in human tissue using immunohistochemistry or mass spectrometry. Additional literature searches were used to determine if a protein was functionally associated with either Aβ or APP in instances where there was no immunohistochemistry evidence of presence in plaques. Key words used in these pubmed searches were: “Alzheimer’s and gene ID” or “Alzheimer’s and protein name”. Plaque enriched proteins identified by mass spectrometry were determined by comparison with Xiong et al*.* [44], which is the only previous study to identify plaque enriched proteins in comparison to non-plaque regions in human brain tissue using mass spectrometry. Published data from Xiong et al*.* was filtered to identify plaque-enriched proteins that were identified by at last 2 peptides, had a fold-change difference of > 1.5 fold between plaques and non-plaques for AD versus controls or preclinical AD versus controls and did not include the word “keratin” or “immunoglobulin” in the protein name to make their data comparable with ours. Proteins with an abundance in the bottom 10% in sAD plaques or preclinical plaques were excluded. Uniprot ID was used to match proteins between studies.

Change in brain protein expression in AD versus controls was determined using our in-house developed database—NeuroPro—which combines results from 33 previous studies that used proteomics to identify consistent protein differences between AD and control human brain tissue [11, 12, 41, 44–73].

### Validation immunohistochemistry

Proteins were selected for validation studies based on the following criteria: enrichment in both EOAD and DS plaques, protein abundance in the top 50% in amyloid plaques, high fold change enrichment in plaques, appropriate commercial antibody available and limited/no previous evidence of presence in plaques by immunohistochemistry. Based on these criteria the following proteins were selected for immunohistochemistry validation studies: MSN, EZR, SMOC1 and ARL8B. 8 µm formalin-fixed paraffin embedded tissue sections containing the hippocampus and surrounding cortex were used for immunohistochemistry validation studies using the fluorescent immunohistochemistry method described above. Primary antibodies used for these validation studies included: MSN (1:200; Proteintech; #16495-1-AP), EZR (1:100; Thermo Scientific; #QG218841), SMOC1 (1:100; Invitrogen; #PA5-31392), ARL8B (1:200; Invitrogen; #PA5-98885), Aβ (combination of 4G8 [BioLegend; #800701] and 6E10 [BioLegend; #803001], both 1:4000). The combined formic acid and citrate buffer antigen retrieval method (described above) was used for all validation immunohistochemistry studies. 63× images of fluorescent immunohistochemistry were collected using a confocal microscope Zeiss 700 with the ZEN Black 2.3 SP1 acquisition software. ARL8B immunoreactivity in neurons, microglia and astrocytes was tested using the same method as above with the following primary antibodies: GFAP (1:1000; BioLegend; #837201), IBA1 (1:200; Millipore; #MABN92-25UG) and MAP2 (1:300, BD Pharmingen, #556320). A negative control was included in all immunohistochemistry experiments, which consisted of a section of AD hippocampal tissue that underwent the same method with the primary antibody omitted.

The percentage of amyloid plaques co-localized with ARL8B or SMOC1 was quantified using whole slide fluorescent scans that were collected using the Aperio VERSA digital slide scanner (Leica) with the 10× objective. Images were visualized and analyzed using the software Aperio ImageScope ver. 12.4.3 (Leica). Plaques co-stained with SMOC1 or ARL8B and Aβ or plaques stained only with Aβ in the hippocampal region were manually counted and then the ratio of co-stained plaques versus total plaques was calculated (co-stained plaques/total plaques × 100). The proportion of the plaques was obtained by plotting total number of plaques compared to the plaques co-stained by SMOC1 or ARL8B and Aβ, using the “grouped” layout of GraphPad Prism 8. Significant differences between groups were identified using one-way ANOVA followed by Tukey multiple comparison’s analysis, using GraphPad Prism 8 software.

## Results

### Differences in Aβ species in EOAD and DS

**Fig. 2:**
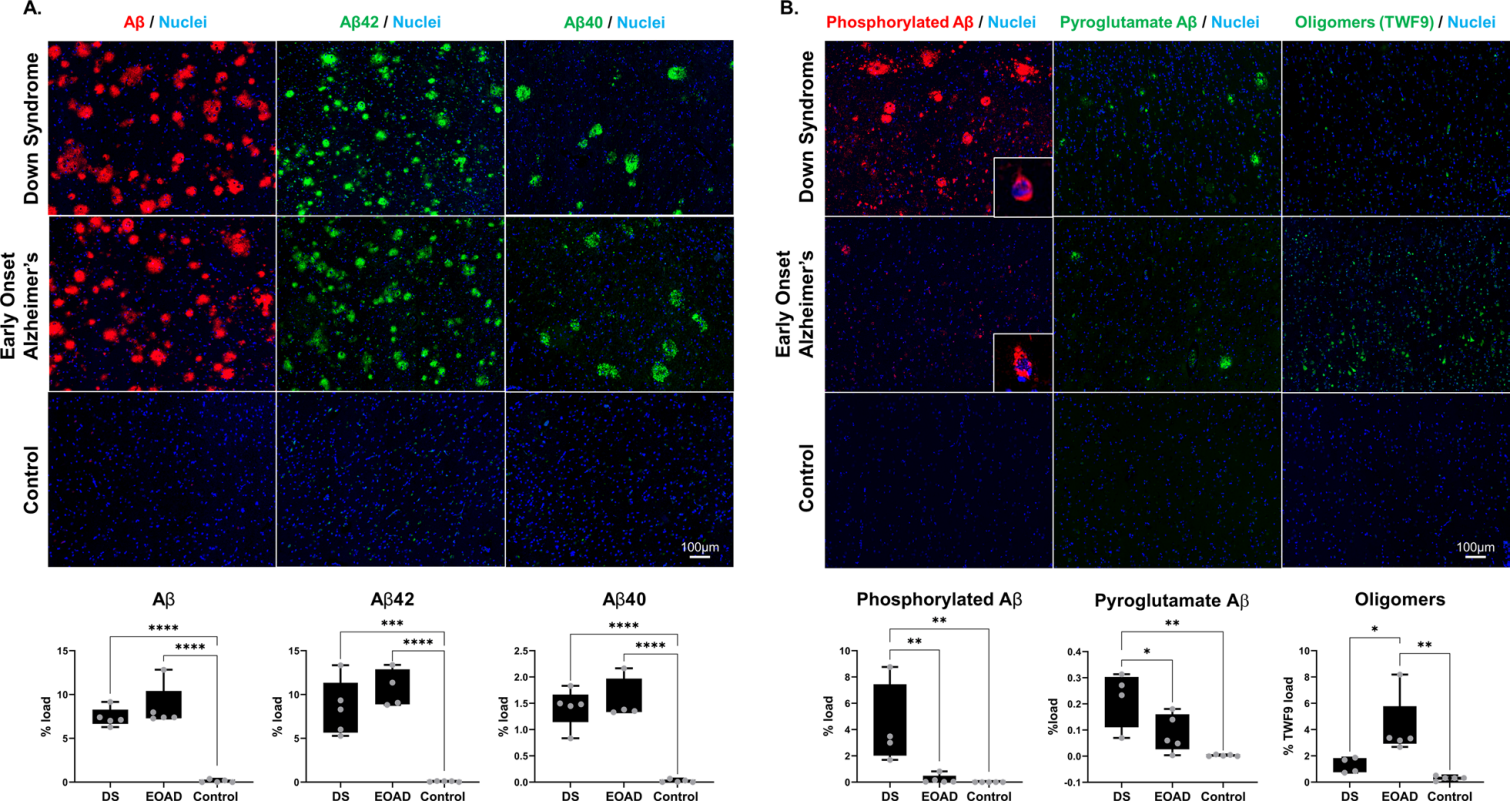
Comparison of levels of different Aβ species and oligomers in DS, EOAD and cognitively normal controls. Representative fluorescent immunohistochemistry images show the distribution of total Aβ, Aβ42, Aβ40, phosphorylated Aβ, pyroglutamate Aβ and oligomers in the cortex. **A** Similar amounts of plaques containing Aβ, Aβ42 and Aβ40 were observed in DS in comparison to EOAD. **B** Phosphorylated Aβ was observed in plaques and intraneuronally in both DS and EOAD. Intraneuronal phosphorylated Aβ was observed in both DS and EOAD (higher magnification image inserts in B). Pyroglutamate Aβ was only observed in plaques in DS and EOAD. Immunostaining for the conformational oligomer antibody TWF9 was observed intraneuronally, but not in plaques. Significantly higher amounts of plaque-associated phosphorylated Aβ and pyroglutamate Aβ were observed in DS in comparison to EOAD and controls. In contrast, significantly higher amounts of oligomers were observed in EOAD in comparison to DS and controls. Significant differences were determined by one-way ANOVA followed by Tukey’s multiple comparisons test. *p < 0.05; **p < 0.01

Amyloid plaques in DS and EOAD had similar amounts of total Aβ, Aβ40 and Aβ42 (Fig. 2A). The size of amyloid plaques was similar in DS and EOAD. However, amyloid plaques in DS had significantly higher amounts of both phosphorylated Aβ and pyroglutamate Aβ than EOAD cases (Fig. 2B). Phosphorylated Aβ immunoreactivity was observed both in plaques and in neurons in DS and EOAD. Two main types of intraneuronal staining were observed: staining consistent with presence in neurofibrillary tangles and neurons containing large puncta of phosphorylated Aβ. Phosphorylated Aβ was also observed in dystrophic neurites. While there were significantly increased levels of phosphorylated Aβ in plaques in DS in comparison to EOAD, similar levels of intraneuronal phosphorylated Aβ were observed in DS and EOAD. Pyroglutamate Aβ was observed in amyloid plaques in both DS and EOAD. Significantly more pyroglutamate Aβ was observed in DS in comparison to EOAD (Fig. 2B).

Oligomers were visualized using the pan-oligomeric antibody TWF9, which is a conformational antibody that recognizes Aβ oligomers in addition to other beta sheet containing oligomers [35]. Consistent with previous studies, TWF9 immunoreactivity was observed in neuronal soma. No immunoreactivity was observed within plaques. DS cases had significantly lower levels of TWF9 immunoreactivity in comparison to EOAD (Fig. 2B).

### Proteomic analysis of EOAD and DS amyloid plaques

Proteomic analysis of plaques and neighboring non-plaque tissue identified 2259 proteins (Additional file 1: Table S1). 85% of proteins (1915 proteins) were identified in both EOAD and DS samples, of which 1355 proteins were identified in all 20 samples, therefore confirming that our proteomic approach is a reliable way to quantify amyloid plaque proteins using microscopic amounts of formalin-fixed paraffin embedded human tissue samples. Proteins present in all 20 samples included major AD-associated proteins such as Aβ, Tau and ApoE, therefore confirming the presence of these proteins both inside plaques and in surrounding non-plaque tissue.

### Proteins enriched in plaques in both EOAD and DS

**Fig. 3:**
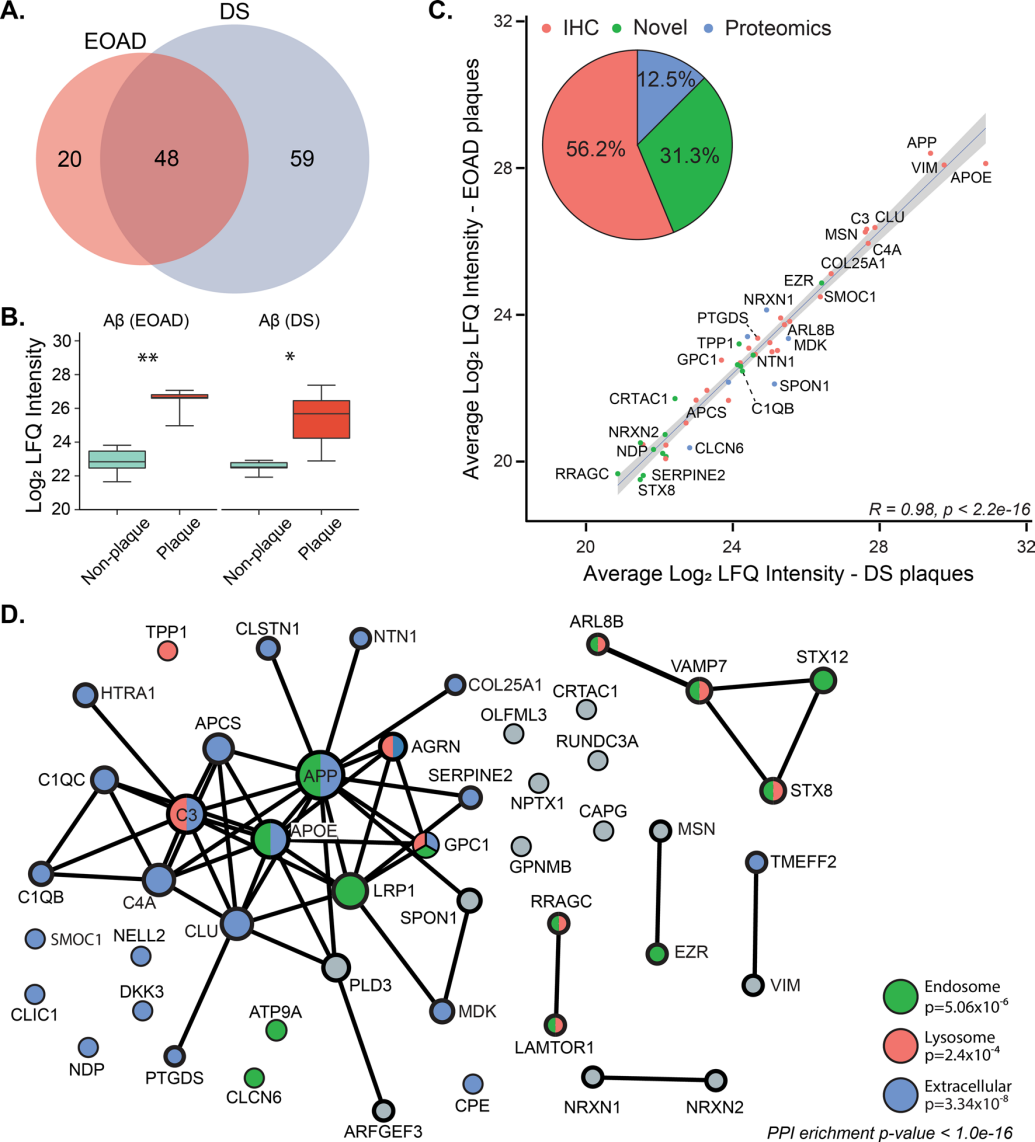
48 proteins were significantly enriched in plaques in both DS and EOAD. **A** 107 proteins were enriched in DS plaques and 68 proteins were enriched in EOAD plaques. Of these, 48 proteins were enriched in both DS and EOAD. **B** Aβ was significantly enriched in plaques in comparison to neighboring non-plaque tissue in both DS (11.92 fold enriched) and EOAD (6.96 fold enriched; paired *t*-test). **C** There was a highly significant correlation in the abundance (determined by intensity values from LC–MS) of common plaque enriched proteins in DS and EOAD. Apolipoprotein E (APOE), APP and vimentin (VIM) were the three most abundant proteins in plaques in both DS and EOAD. Proteins are coloured to show if each is a previously validated plaque protein (red: 56.2% proteins previously validated as a plaque protein in a targeted immunohistochemistry [IHC] study; blue: 12.5% proteins previously validated as a plaque protein in a proteomics study only) or a novel identified plaque protein (green; 31.3% proteins). **D** Pathway analysis of the 48 proteins commonly enriched in plaques in both DS and EOAD showed a highly significant degree of protein–protein interactions (*p* < 1.0 × 10^−16^). Pathway analysis showed that these proteins were highly enriched extracellular proteins (blue), endosome proteins (green) or lysosome proteins (red). *p < 0.05; **p < 0.01

The main aim of this study was to identify proteins that were enriched in amyloid plaques in EOAD and DS in comparison to surrounding non-plaque tissue. 127 proteins were significantly enriched in amyloid plaques in either EOAD or DS (Additional file 1: Table S1). 48 proteins were consistently enriched in both DS and EOAD plaques (Table 2, Fig. 3). Systematic literature searches revealed that 33/48 proteins have been previously confirmed as amyloid plaque proteins in late-onset AD, therefore validating our mass spectrometry approach and providing new evidence that similar proteins are enriched in amyloid plaques in different subtypes of AD (Table 2). In addition, we identified 15 proteins that were enriched in plaques in both EOAD and DS (Table 2) that were not previously known to be plaque associated proteins. Four of these proteins have been previously associated with either Aβ or APP. Here, we provide the first evidence that these proteins are enriched in amyloid plaques. The remaining 11 proteins are amyloid plaque proteins that have not been previously associated with Aβ, APP or amyloid plaques in any subtype of AD (Table 2).

**Table 2:** 48 proteins consistently enriched in plaques in EOAD and DS

Uniprot	Gene	Protein	Enrichment in EOAD plaques (fold change)	Enrichment in DS plaques (fold change)	Known plaque protein?	Difference in AD brain tissue	Mediates Aβ pathology?
Previously confirmed plaque proteins—immunohistochemistry							
Q9BXS0	COL25A1	Collagen alpha-1	104.3	113.1	Yes [74]	Increased	Increases pathology [6, 75]
O95631	NTN1	Netrin-1	34.9	58.7	Yes [60]	Increased	Decreases pathology [76]
P21741	MDK	Midkine	31.4	70.4	Yes [77]	Increased	Decreases pathology [78]
Q92743	HTRA1	Serine protease HTRA1	19.0	42.8	Yes [79]	Increased	Decreases pathology [80]
Q9H4F8	SMOC1	SPARC-related modular calcium-binding protein 1	12.9	58.8	Yes [60]	Increased	Unknown
P02649	APOE	Apolipoprotein E	10.4	17.2	Yes [81]	Increased	Increases pathology [82, 83]
Q14956	GPNMB	Transmembrane glycoprotein NMB	7.8	17.8	Yes (in plaque-associated microglia) [84]	Increased	Unknown
P0C0L4	C4A	Complement C4-A	7.5	10.1	Yes [85]	Increased	Unknown
P35052	GPC1	Glypican-1	7.5	8.5	Yes [86]	Decreased	Increases pathology [87]
P02743	APCS	Serum amyloid P-component	4.9	10.8	Yes [88]	Increased	Increases pathology [89]
Q9UIK5	TMEFF2	Tomoregulin-2	4.8	6.9	Yes [90]	N/a	Decreases pathology [91]
P02746	C1QB	Complement C1q subcomponent subunit B	3.3	4.3	Yes [92]	N/a	Increases pathology [93, 94]
P10909	CLU	Clusterin	3.2	4.0	Yes [95]	Increased	Increases pathology [7, 96]
Q00604	NDP	Norrin	2.9	4.6	Yes [79]	Increased	Unknown
P05067	APP	Amyloid-beta precursor protein	2.8	5.9	Yes [97]	Increased	Increases pathology [98]
P02747	C1QC	Complement C1q subcomponent subunit C	2.7	8.4	Yes [92]	Increased	Increases pathology [93, 94]
P01024	C3	Complement C3	2.5	2.9	Yes [92]	Increased	Increases pathology [94, 99, 100]
P41222	PTGDS	Prostaglandin-H2 D-isomerase	2.2	3.0	Yes [101]	Increased	Decreases pathology [101]
P26038	MSN	Moesin	2.1	2.6	Yes, in plaque-associated microglia [102]	Increased	Decreases pathology [103]
P07093	SERPINE2	Glia-derived nexin	2.1	4.3	Yes [104]	Decreased	Increases pathology [105, 106]
Q9UBP4	DKK3	Dickkopf-related protein 3	2.1	1.8	Yes [107]	Increased	Decreases pathology [108]
Q8IV08	PLD3	Phospholipase D3	2.0	2.0	Yes [109]	N/a	Decreases pathology [110, 111]
O00468	AGRN	Agrin	1.9	2.9	Yes [112]	Increased	Decreases pathology [113]
Q07954	LRP1	Prolow-density lipoprotein receptor-related protein 1	1.8	2.1	Yes [114]	Increased	Inconsistent effects on pathology [115]
P08670	VIM	Vimentin	1.7	1.8	Yes, in surrounding astrocytes [116]	Increased	Increases pathology [117]
P16870	CPE	Carboxypeptidase E	1.6	2.1	Yes [118]	Increased	Unknown
Q15818	NPTX1	Neuronal pentraxin-1	1.6	1.7	Yes [119]	Increased	Increases pathology [120]
Previously confirmed plaque protein—proteomics							
Q9NRN5	OLFML3	Olfactomedin-like protein 3	19.2	18.9	Yes [44]	Increased	Unknown
Q9HCB6	SPON1	Spondin-1	6.9	16.5	Yes [44]	N/a	Decreases pathology [121, 122]
O94985	CLSTN1	Calsyntenin-1	5.4	8.1	Yes [44]	Decreased	Increases pathology [123]
Q9ULB1	NRXN1	Neurexin-1	2.9	2.8	Yes [44]	Increased	Unknown
P51797	CLCN6	Chloride transport protein 6	2.8	9.7	Yes [44]	Increased	Unknown
Q9NVJ2	ARL8B	ADP-ribosylation factor-like protein 8B	2.2	2.9	Yes [44]	Increased	Decreases pathology [124]
Novel plaque proteins—mechanistic link with Aβ or APP							
O75110	ATP9A	Probable phospholipid-transporting ATPase IIA	1.8	2.3	No, but associated with Aß [125]	Increased	Increases pathology [125]
P15311	EZR	Ezrin	1.7	2.6	No, but associated with APP [103]	Increased	Decreases pathology [103]
O00299	CLIC1	Chloride intracellular channel protein 1	1.6	1.7	No, but associated with Aß [126]	Increased	Increases pathology [126]
O14773	TPP1	Tripeptidyl-peptidase 1	1.6	2.1	No, but associated with Aß [127]	Increased	Decreases pathology [127]
Novel plaque proteins—no previous association with Aβ or APP							
P51809	VAMP7	Vesicle-associated membrane protein 7	3.0	4.0	No	N/a	Unknown
Q9UNK0	STX8	Syntaxin-8	3.2	2.4	No	Increased	Unknown
Q5TH69	ARFGEF3	Brefeldin A-inhibited guanine nucleotide-exchange protein 3	3.2	5.2	No	Increased	Unknown
Q6IAA8	LAMTOR1	Ragulator complex protein LAMTOR1	2.6	2.9	No	N/a	Unknown
Q59EK9	RUNDC3A	RUN domain-containing protein 3A	2.3	5.6	No	N/a	Unknown
P40121	CAPG	Macrophage-capping protein	2.2	1.9	No	Increased	Unknown
Q9NQ79	CRTAC1	Cartilage acidic protein 1	2.1	2.2	No	N/a	Unknown
Q9P2S2	NRXN2	Neurexin-2	1.9	2.5	No	N/a	Unknown
Q99435	NELL2	Protein kinase C-binding protein NELL2	1.8	3.9	No	N/a	Unknown
Q9HB90	RRAGC	Ras-related GTP-binding protein C	1.9	2.2	No	N/a	Unknown
Q86Y82	STX12	Syntaxin-12	1.5	2.0	No	N/a	Unknown

Proteins listed in order of fold change enrichment in EOAD; separated into previously confirmed plaque proteins, associated with Aβ or APP, and novel. “Previously confirmed plaque proteins” were determined by published immunohistochemistry evidence of protein presence in plaque or by > 1.5 fold enrichment in plaque in comparison to neighboring non-plaque tissue in late onset AD or preclinical AD [44]. Difference in AD tissue was determined by comparison with 33 previous proteomic studies of human AD brain tissue. “Mediates Aβ pathology?” determined by literature searches for “Alzheimer’s disease and gene ID or protein name”. Protein was designated as mediating Aβ pathology if altering protein expression in transgenic animal models or cell culture affected amyloid pathology

As expected, Aβ was highly enriched in plaques in comparison to the surrounding non-plaque tissue (12 and sevenfold enriched in EOAD and DS plaques respectively; Fig. 3B). In contrast, while tau was abundant in both plaques and neighboring non-plaque tissue in DS and EOAD, there was no evidence of enrichment of tau in amyloid plaques. Examination of the abundance (overall intensity in plaques) of the 48 proteins enriched in both EOAD and DS showed that the most abundant proteins present were well-known plaque proteins (e.g. APP, ApoE, vimentin, clusterin, complement C3 and complement C4a; Fig. 3C). We also observed a very high correlation in the total concentration of these proteins in plaques between EOAD and DS (Fig. 3C). The most abundant novel plaque protein in both DS and EOAD was ezrin (EZR), which was one of the proteins selected for immunohistochemistry validation studies (Fig. 3C).

**Fig. 4:**
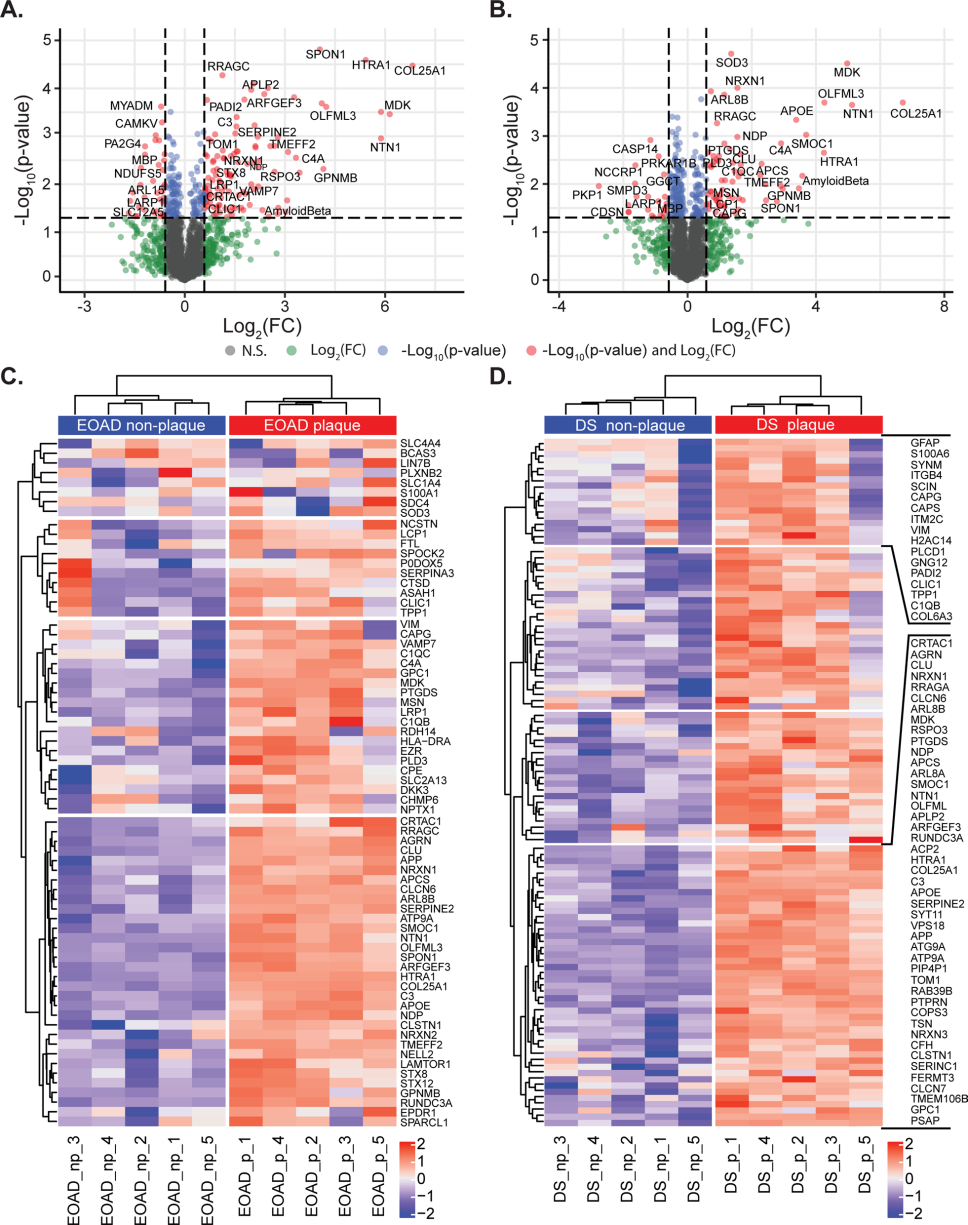
Significantly altered proteins in plaques in comparison to neighboring non-plaque tissue. **A**, **B** Volcano plots highlight proteins in red that were significantly altered in plaques in comparison to non-plaque tissue. Significance was determined by a combination of *p* < 0.05 and a fold change difference of greater than 1.5 fold. Proteins that have a fold change difference of greater than 1.5 fold only are shown in green and proteins that had a difference of *p* < 0.05 only are shown in blue. The total number of proteins included in the analysis was 2059 proteins for DS and 2115 proteins for EOAD. Proteins are identified by gene IDs. **C**, **D** Unsupervised clustering heatmaps for proteins that were significantly altered in DS or EOAD. Plaque and non-plaque samples independently clustered, highlighting the significantly different protein expression between plaque and non-plaque samples for DS and EOAD. All gene IDs are indicated for EOAD in each row whilst only genes from cluster 1 and 4 are marked for DS, constituting a divergent cluster and highly enriched cluster of genes respectively for DS plaques

Examination of the proteins that had the highest enrichment in plaques in both DS and EOAD included many proteins less studied in the AD field (Table 2; Additional file 1: Table S3). For example, COL25A1 was the most highly enriched protein in plaques in both EOAD and DS (104 and 113-fold enriched respectively). Other highly enriched plaque proteins in both EOAD and DS included MDK, NTN1, HTRA1, SMOC1 and OLFML3 (Fig. 4A, B). The 48 proteins consistently enriched in plaques in both EOAD and DS also showed a highly significant degree of protein–protein interaction (*p* < 1.0 × 10^–16^; Fig. 3D) and were almost exclusively classified as either vesicle (enrichment FDR: 4.32 × 10^−9^) or extracellular proteins (enrichment FDR: 3.34 × 10^−8^). The enrichment of vesicle proteins was predominantly driven by endosome or lysosome proteins (Fig. 3D; Additional file 1: Table S3). Synapse proteins were also particularly enriched (enrichment FDR: 1.90 × 10^−3^).

### Differences in plaque enriched proteins in EOAD and DS

Our results suggest that that major plaque enriched proteins in EOAD and DS were largely the same. The consistency of protein enrichment in plaques was even noted at an individual case level (Fig. 4C, D). However, we were interested to determine whether there was evidence of plaque protein enrichment that was unique to either DS or EOAD beyond these common plaque-enriched proteins. 20 proteins were uniquely enriched in plaques in EOAD (Additional file 1: Table S4) and 59 proteins were uniquely enriched in plaques in DS (Additional file 1: Table S5). Pathway analysis of proteins that were uniquely enriched in plaques in either DS or EOAD showed that these proteins were also enriched in endosomal or lysosomal proteins, similar to the commonly enriched plaque proteins. These protein differences between DS and EOAD did not suggest the presence of unique disease mechanisms driving plaque development in DS or EOAD: pathway analysis showed that these proteins did not cluster to a particular functional pathway and the majority of proteins showed the same trend for enrichment in plaques in the other group. 80% (63/79 proteins) of proteins uniquely enriched in plaques in either EOAD or DS were still increased in plaques in the other subtype of AD, albeit at a level that did not meet our criteria for ‘enrichment in plaques’. Therefore, these results suggest that largely the same proteins are enriched in amyloid plaques in EOAD and DS.

We also directly compared plaque protein levels in DS and EOAD. For this analysis, plaque protein levels that were normalized against background protein levels for each individual case were used. 38 proteins were significantly different between DS and EOAD plaques after correction for background protein differences. 25 proteins were significantly higher and 13 proteins were significantly lower in DS plaques in comparison to EOAD plaques (Additional file 1: Table S7). Pathway analysis did not highlight enrichment of any cellular compartments or pathways for significantly different proteins in DS and EOAD plaques. Again, suggesting that plaque protein composition was largely the same in DS and EOAD.

We also examined if the triplication of chromosome 21 in DS resulted in any major differences in plaque associated proteins. Our proteomic analysis identified 22 proteins with genes on chromosome 21 (Additional file 1: Table S6). Of these, only three proteins were enriched in plaques in DS: APP, ITGB2 and COL18A1. APP was commonly enriched in plaques in both EOAD and DS. While ITGB2 and COL18A1 both had higher levels in plaques in comparison to non-plaques in EOAD, their level did not meet our criteria for designation as “enriched”. Therefore, our results suggest that the triplication of chromosome 21 is not necessarily associated with enrichment of those gene products in plaques, but rather may enhance the enrichment of selected proteins in plaques.

### Validation: comparison with previous proteomic studies

**Fig. 5:**
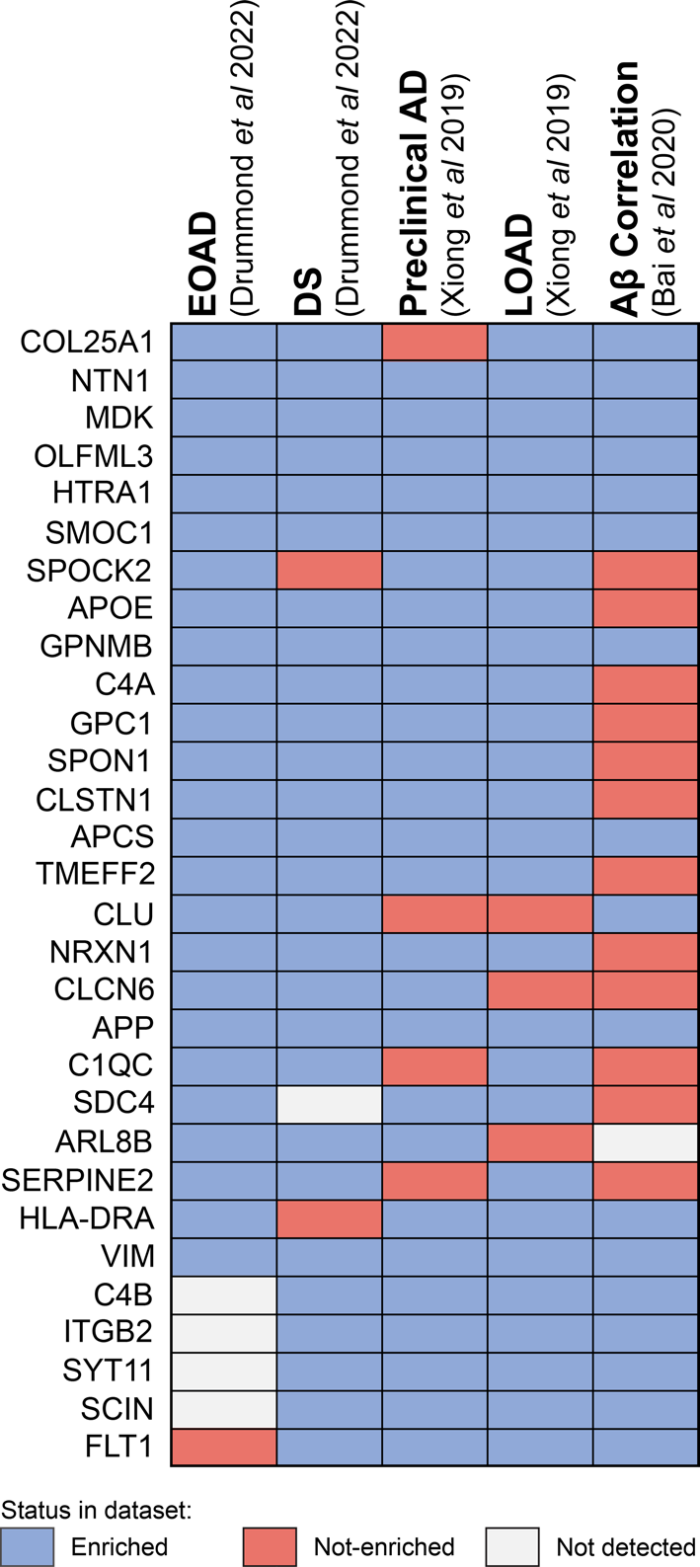
Comparison of common plaque enriched proteins in DS and EOAD with previous proteomic studies. Plot shows the 30 plaque proteins that were either identified in plaques or correlated with Aβ in at least 3 previous proteomic studies. Proteomic data was obtained from [44] for enrichment in preclinical AD or LOAD plaques and [60] for correlation with Aβ. Blue boxes indicate protein significantly enriched in plaques in comparison to surrounding non-plaque tissue or significantly correlated with Aβ. Red boxes indicate detection in the study but no enrichment in plaques or correlation with Aβ. White boxes indicate instances when a protein was not detected. Proteins are listed in order of fold change enrichment in plaques in EOAD followed by fold change enrichment in DS plaques if not enriched in EOAD

Only one prior study has examined the proteome of amyloid plaques in comparison to surrounding non-plaque tissue [44]. This study identified proteins that were enriched in amyloid plaques in late-onset AD and preclinical AD. Despite the power of their dataset being limited by a small sample size (n = 3 cases/group, pooled prior to mass spectrometry) and the different subtypes of AD analyzed in their study in comparison to ours, we were pleased to see that many of our plaque enriched proteins were validated in this previous study. 43 proteins were identified in both our study and enriched in late-onset AD plaques in Xiong et al. 26/43 commonly identified proteins were significantly enriched in either DS or EOAD plaques (Additional file 1: Table S1). The majority of the remaining proteins were also increased in plaques in our study, however they did not reach the criteria for significance in our study. All of the top 10 most highly enriched proteins in plaques in DS and EOAD in our study were also enriched in plaques in late onset AD (Fig. 5).

Xiong et al*.* also identified 78 proteins that were enriched in plaques in preclinical AD. 53 of these proteins were also identified in our study, of which, 30 were enriched in DS or EOAD plaques (Additional file 1: Table S1). The most notable protein that was not enriched in preclinical AD plaques was COL25A1, which was the most highly enriched protein in both DS and EOAD plaques in our study and was enriched in late-onset AD plaques in Xiong et al. [44]. This suggests that COL25A1 may only become enriched in plaques at a later stage of disease development. In contrast, the remaining top 10 most enriched proteins for both DS and EOAD were also enriched in plaques in preclinical AD (Fig. 5), suggesting that plaques in preclinical AD largely contain the same proteins present in plaques at advanced stages of AD.

We also compared our data to Bai et al. [60] who identified 28 proteins that correlated with Aβ abundance in human brain tissue throughout the progression of AD. 20 of these proteins were also identified in our study, of which 13 were significantly enriched in DS and/or EOAD plaques (Additional file 1: Table S1). The remaining 7 proteins were also increased in DS and/or EOAD plaques, however these did not reach our statistical stringency level to be considered a plaque-enriched protein.

The combined analysis of our data with these two previous studies identified 30 proteins that were consistently enriched in plaques or correlated with Aβ in at least 3 analyses (Fig. 5). This group of proteins represent a consistent amyloid plaque signature highlighting proteins that likely have an important role in amyloid plaque pathology in addition to Aβ. While the some of these proteins are well known plaque proteins (e.g. APP, ApoE, clusterin), the role of many of these proteins in AD is comparatively much less studied including 8 proteins that have only been discovered as an amyloid plaque protein in proteomic studies (OLFML3, SPON1, CLSTN1, NRXN1, CLCN6, ARL8B, SYT11, SCIN). Combined, these comparisons with previous studies validates our findings and provides additional evidence that amyloid plaques are enriched in many proteins in addition to Aβ, many of which are likely to be of pathological importance in AD and merit further investigation.

### Validation: immunohistochemistry

**Fig. 6:**
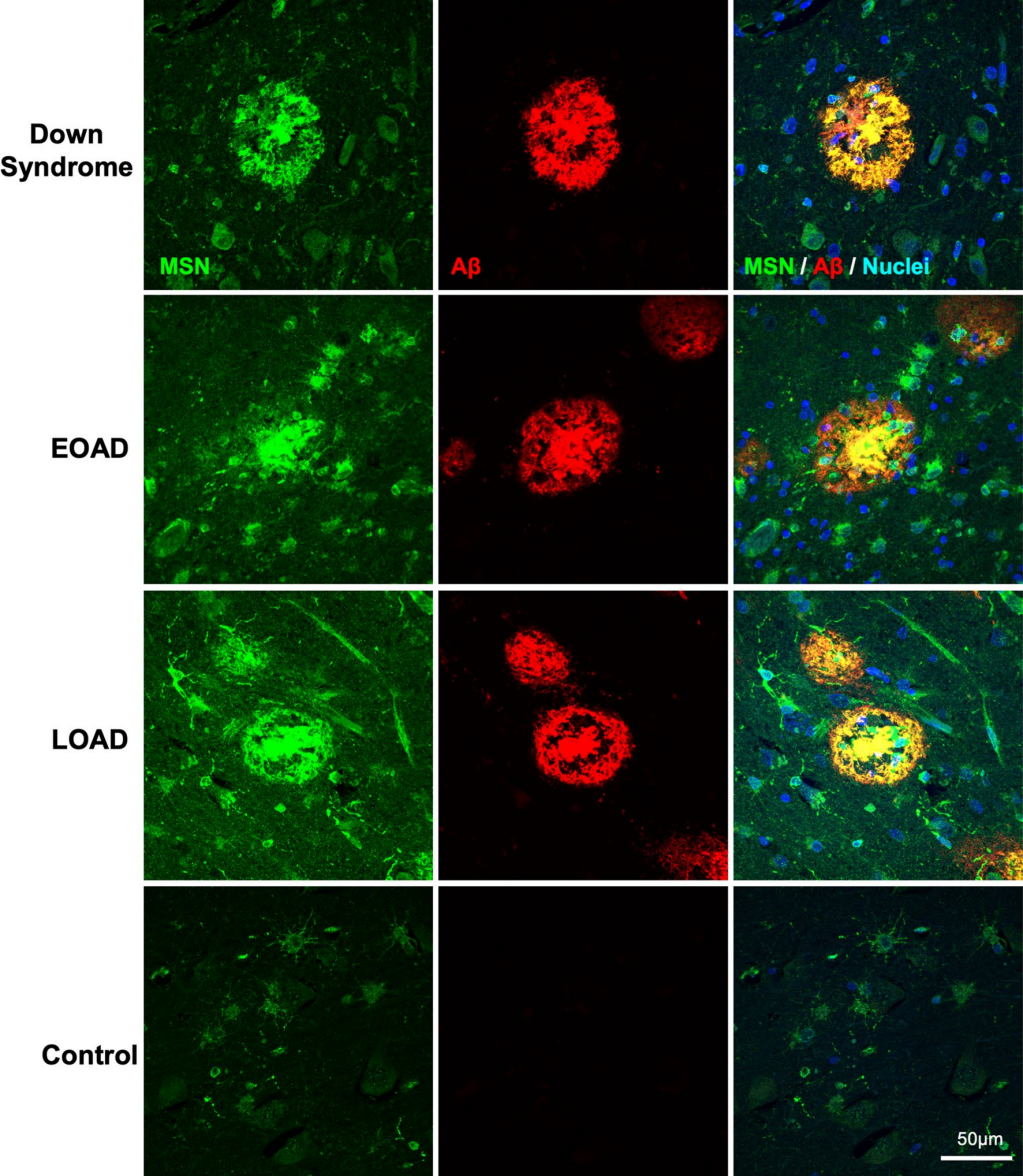
Validation of moesin as a plaque enriched protein in human brain tissue by immunohistochemistry. Enrichment of moesin (MSN) in amyloid plaques was observed in DS, EOAD and LOAD cases. Moesin was also observed outside of plaques in all tissue examined (including cognitively normal control tissue) in cells consistent with a microglial morphology

**Fig. 7:**
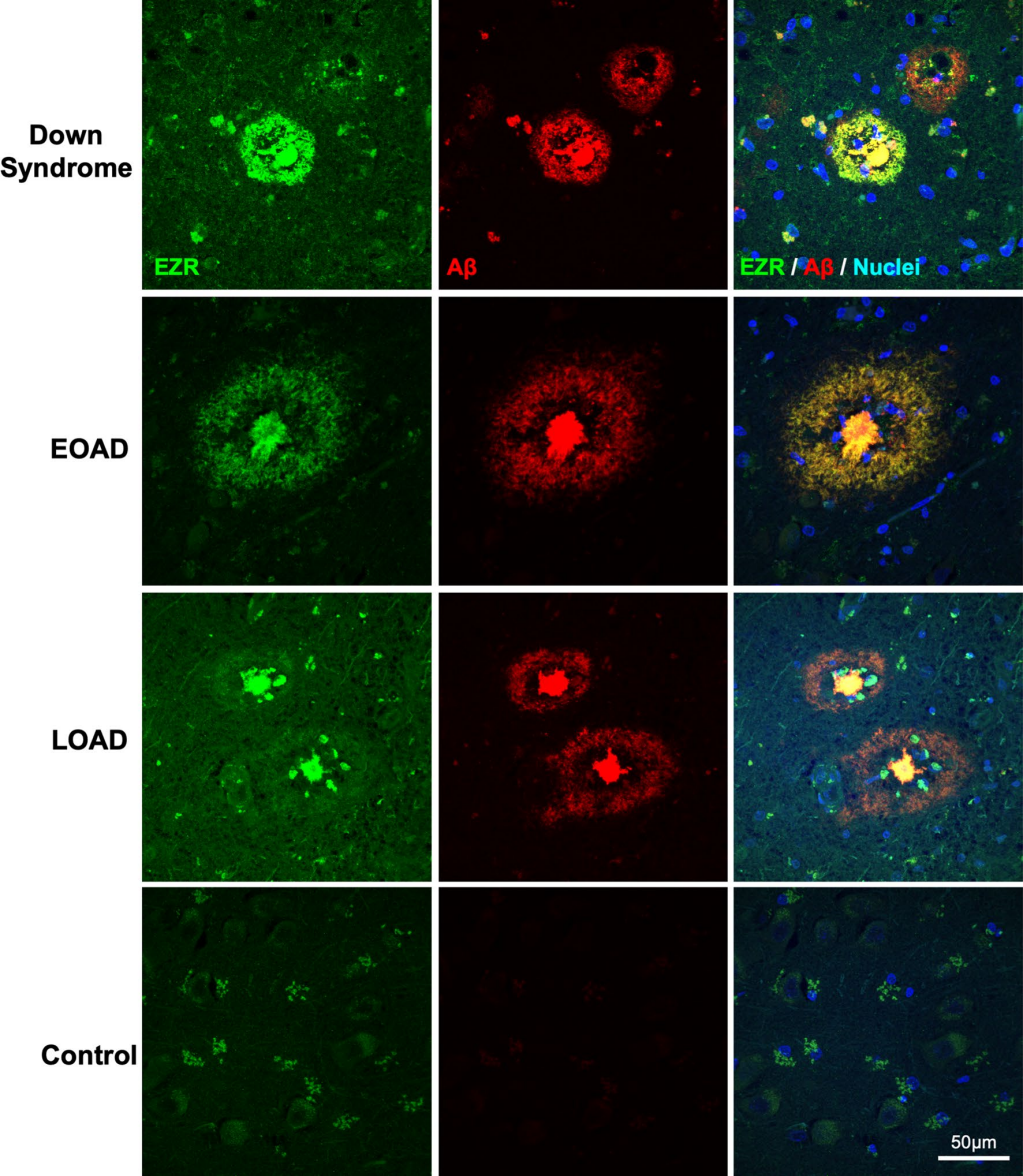
Validation of ezrin as a plaque enriched protein in human brain tissue by immunohistochemistry. Enrichment of ezrin (EZR) was observed in amyloid plaques in DS, EOAD and LOAD cases

**Fig. 8:**
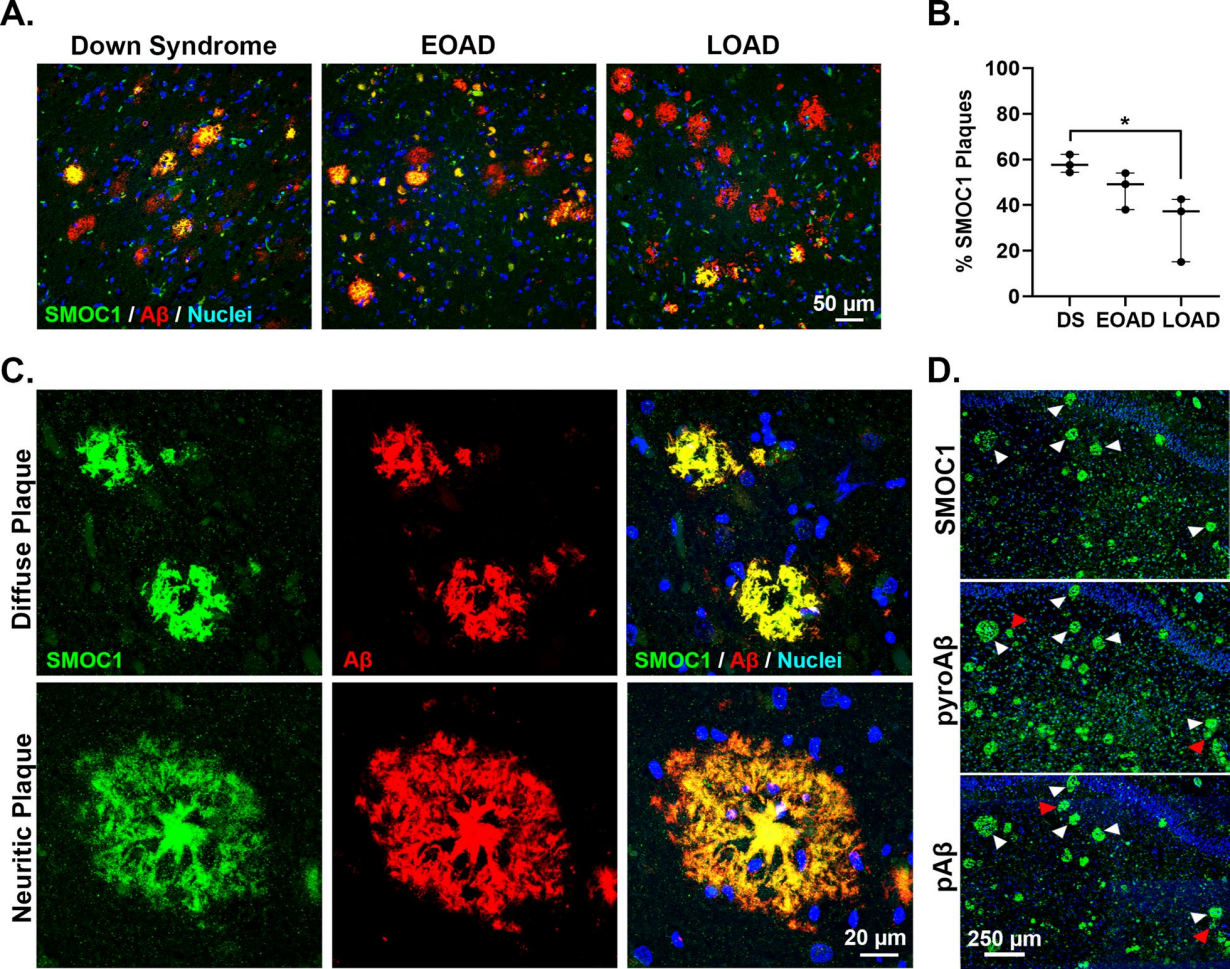
Validation of SMOC1 as a plaque enriched protein in human brain tissue by immunohistochemistry. **A** Enrichment of SMOC1 was observed in a sub-population of amyloid plaques in DS, EOAD and LOAD cases. **B** Plot shows percentage of SMOC1 immunoreactive plaques in the hippocampus of DS, EOAD and LOAD cases (n = 3/group). Results generated by an analysis of 321 ± 47 hippocampal plaques (average ± SEM) in each case. The ratio of SMOC1 positive plaques (immunoreactive for both Aβ and SMOC1) over the total number of amyloid plaques was calculated for each case in DS, EOAD and LOAD. **C** Representative images of diffuse and neuritic plaques immunolabeled with SMOC1. **D** Representative images of SMOC1, pyroglutamate Aβ and phosphorylated Aβ immunolabelled plaques in the hippocampus of a representative Down syndrome case. Fluorescent immunohistochemistry was used to identify SMOC1, pyroglutamate Aβ or phosphorylated Aβ immunoreactive plaques on three sequential hippocampal sections from the same case. White arrowheads show SMOC1 immunoreactive amyloid plaques that were also immunoreactive for pyroglutamate Aβ and phosphorylated Aβ species. Red arrowheads show pyroglutamate Aβ and/or phosphorylated Aβ immunoreactive plaques negative for SMOC1. *p < 0.05

Fluorescent immunohistochemistry was used to validate the enrichment of four proteins in amyloid plaques. Ezrin (EZR) was selected as it was the most abundant novel plaque protein identified in our study. ARL8B was selected as a representative plaque-enriched lysosomal protein that had no prior immunohistochemistry evidence of presence in amyloid plaques. Moesin (MSN) and SMOC1 were selected as both have only one prior publication confirming their presence in plaques using immunohistochemistry, but no immunohistochemistry evidence of enrichment in plaques in EOAD or DS. Fluorescent immunohistochemistry confirmed that ezrin, moesin, ARL8B and SMOC1 were enriched in amyloid plaques in comparison to surrounding non-plaque tissue in DS, EOAD and late-onset sporadic AD. Moesin (Fig. 6), Ezrin (Fig. 7), and SMOC1 (Fig. 8) strongly co-localized with Aβ in amyloid plaques. Particularly intense immunoreactivity was observed in the aggregated core of dense-cored plaques for these proteins. Moesin was also observed in cells with a microglial morphology in both AD and control cases, consistent with a previous study that confirmed that moesin is a microglial protein [102].

SMOC1 strongly co-localized with amyloid fibrils only in a subset of amyloid plaques (Fig. 8). The proportion of SMOC1 immunoreactive plaques in the hippocampus varied between subtypes of AD; SMOC1 was present in 58% amyloid plaques in DS in comparison to 47% of plaques in EOAD and 32% of plaques in late-onset AD (Fig. 8A, B). This was consistent with our proteomic results that found a greater enrichment of SMOC1 in DS plaques in comparison to EOAD plaques. Both neuritic and diffuse plaques showed SMOC1 immunoreactivity (Fig. 8C). Qualitatively, the proportion of SMOC1 immunoreactive plaques was higher in the hippocampus than in the neighboring cortex in all subtypes of AD. Interestingly, there was a large amount of colocalization of SMOC1 with plaques that also contained post-translationally modified Aβ species (white arrows, Fig. 8D). Minimal basal SMOC1 staining was observed in age-matched control cases, with the most consistent basal SMOC1 expression present in localized pockets of the choroid plexus.

**Fig. 9:**
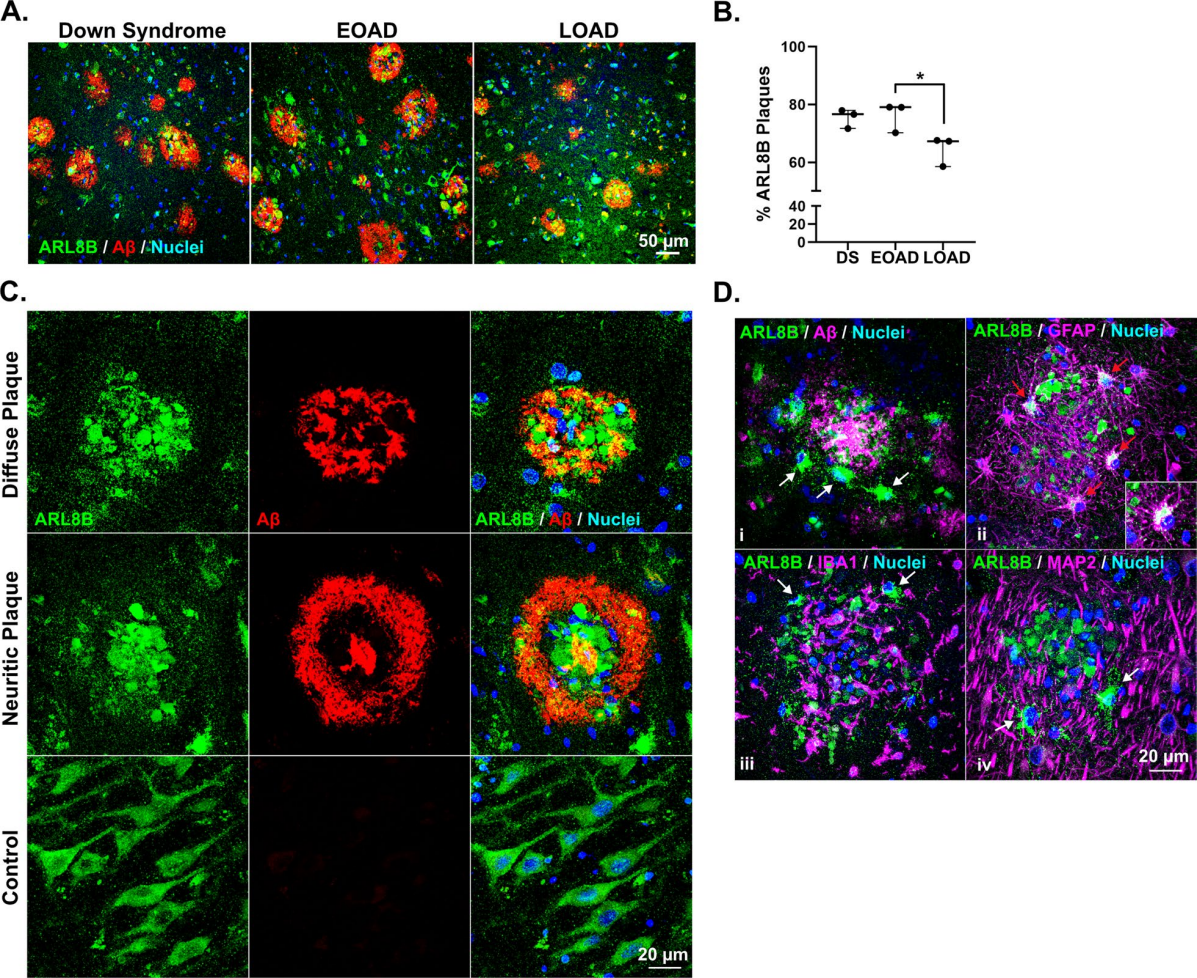
Validation of ARL8B as a plaque enriched protein in human brain tissue by immunohistochemistry. **A** Enrichment of ARL8B in amyloid plaques was observed in DS, EOAD and LOAD cases. **B** Plot shows percentage of ARL8B immunoreactive plaques in the hippocampus of DS, EOAD and LOAD cases (n = 3/group). Results generated by an analysis of 309 ± 41 hippocampal plaques (average ± SEM) in each case. The ratio of ARL8B positive plaques (immunoreactive for both Aβ and ARL8B) over the total number of amyloid plaques was calculated for each case in DS, EOAD and LOAD. **C** Representative images showing ARL8B distribution in amyloid plaques. Bright puncta of ARL8B were diffusely present throughout both diffuse and neuritic plaques. Basal ARL8B staining was observed in controls in neuron soma. **D** Intense ARL8B immunoreactivity was observed in plaque-associated cells (i; arrows). Double-fluorescent immunohistochemistry showed that these plaque-associated cells with intense ARL8B immunoreactivity were a subset of plaque-associated reactive astrocytes (ii; GFAP, red arrows), and not plaque associated reactive microglia (iii; IBA1, white arrows) or neurons (iv; MAP2, white arrows). Insert in ii shows a higher magnification image of the colocalization of ARL8B and GFAP in plaque associated astrocytes

ARL8B was also abundant in amyloid plaques in all subgroups (Fig. 9). In contrast to SMOC1, the proportion of ARL8B immunoreactive plaques in the hippocampus was similar in DS and EOAD (77% and 79%, respectively; Fig. 9A, B). However, a significantly lower proportion of plaques contained ARL8B in late-onset AD in comparison to EOAD (Fig. 9A, B). Two distinct patterns of plaque-associated ARL8B staining were observed. In one subset of amyloid plaques, bright puncta of ARL8B were diffusely present throughout plaques (Fig. 9C). In these plaques, ARL8B did not strongly colocalize with Aβ. Instead, ARL8B was often observed in the regions of amyloid plaques that were not brightly stained for Aβ (Fig. 9C). Qualitatively, ARL8B colocalization in amyloid plaques was more commonly observed in the hippocampus than the cortex. Basal ARL8B staining in control hippocampal sections was observed in neuron soma throughout the cytoplasm and occasionally in primary processes (Fig. 9C). Staining was particularly bright in hippocampal pyramidal neurons. Abundant ARL8B was also observed in granule cells in the dentate gyrus, in the choroid plexus, and in the nucleus of some cells in white matter. The same pattern of basal staining was observed in controls and all subtypes of AD. In the second subset of amyloid plaques, intense ARL8B immunoreactivity was observed in specific plaque-associated cells (Fig. 9D). These cells were located at the periphery of plaques and had bright, punctate ARL8B throughout the cell cytoplasm and primary processes (Fig. 9D) and had morphology consistent with reactive glia. Double fluorescent immunohistochemistry against ARL8B and MAP2, IBA1, or GFAP showed that these ARL8B positive plaque-associated cells were a subset of reactive plaque associated astrocytes.

**Fig. 10:**
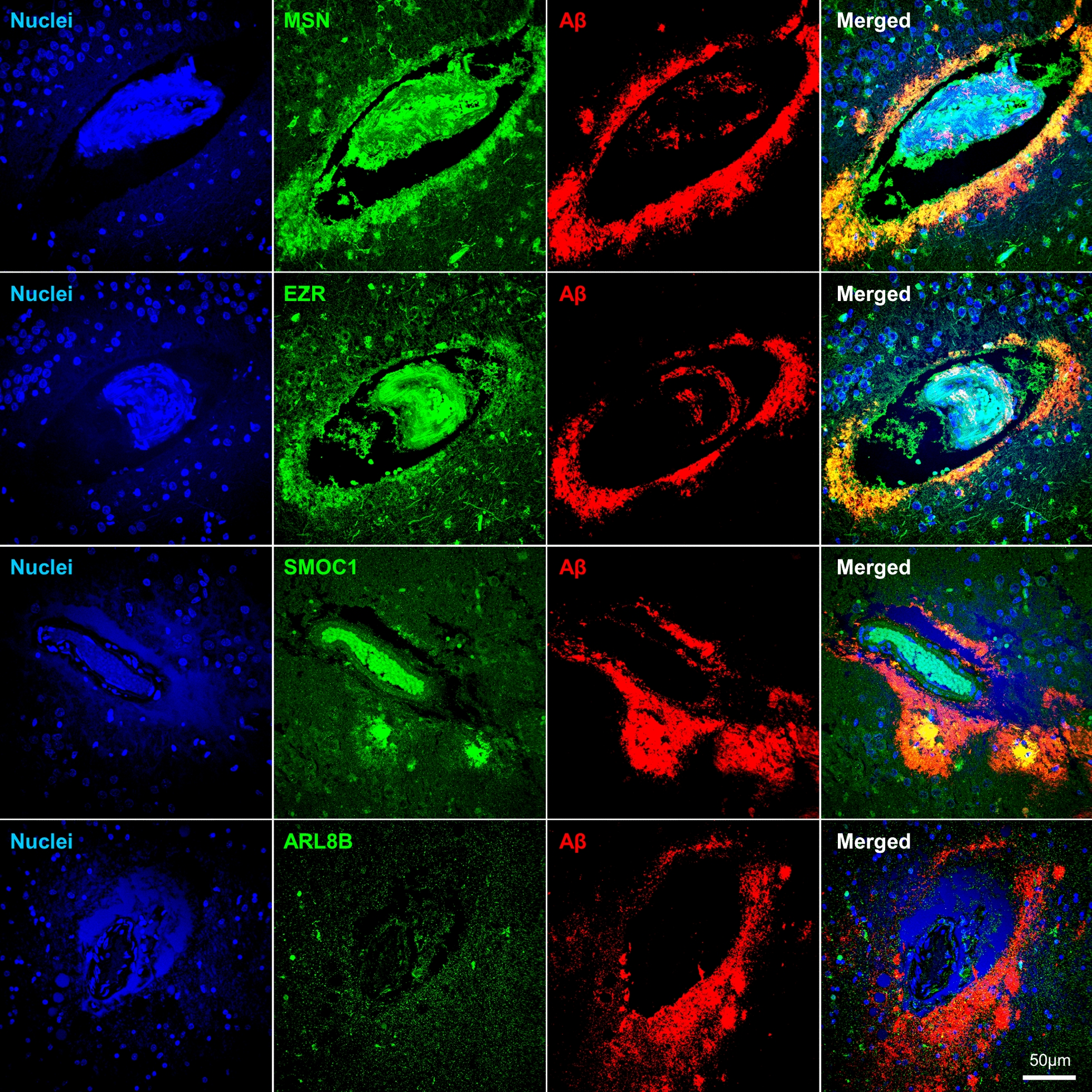
Co-localization of plaque enriched proteins with vascular amyloid deposition. Representative images of vascular amyloid pathology immunolabeled with moesin (MSN), ezrin (EZR), SMOC1, ARL8B (green) and Aβ (4G8/6E10, red). Moesin, ezrin and SMOC1 co-localized with vascular amyloid pathology while ARL8B did not

We also validated the presence or absence of these four plaque proteins in vascular amyloid pathology. MSN, EZR and SMOC1 immunoreactivity occasionally co-localized with CAA or in plaques which were in direct contact with blood vessels. However, ARL8B immunoreactivity was absent in vascular amyloid pathology, which is consistent with its weak direct colocalization with Aβ in amyloid plaques (Fig. 10).

## Discussion

Our results show that amyloid plaques in DS and EOAD are highly enriched in many proteins in addition to Aβ. Here, we have identified a core group of 48 proteins that are consistently enriched in plaques in comparison to neighboring non-plaque tissue in DS and EOAD. Many of these enriched plaque proteins have been validated in previous studies to colocalize with plaques or correlate with Aβ pathology in typical late onset AD, suggesting that this core group of enriched plaque proteins is consistent in both early and late onset AD subtypes. We also identified 15 novel proteins that were consistently enriched in plaques in both DS and EOAD. Our immunohistochemistry studies showed that while similar proteins are present in plaques in DS and EOAD the relative abundance of some of these proteins (e.g. pyroglutamate Aβ, phosphorylated Aβ, SMOC1) is distinct in plaques in DS and EOAD.

Our unbiased proteomics approach highlighted the striking enrichment of many proteins in amyloid plaques that have not been extensively studied in the context of AD such as COL25A1, SMOC1, NTN1, MDK, OLFML3 and HTRA1. The small number of previous studies examining the role of these proteins in AD suggest that these proteins likely have an important role in AD pathology. All of these highly enriched plaque proteins were also enriched in amyloid plaques in typical late onset AD [44] and 5/6 of these proteins were enriched in plaques in preclinical stages of AD [44], suggesting a possible role in the development of early AD pathology. Proteomic studies of human AD brain bulk tissue homogenate showed that all 6 highly enriched plaque proteins were increased in AD brain tissue in multiple brain regions in comparison to age-matched cognitively normal control brain tissue [53–55, 57, 62, 128]. Prior studies have shown that COL25A1 expression increases Aβ pathology, while NTN1, MDK and HTRA1 decreases Aβ pathology in either mouse models or cell models of AD [75, 76, 80, 129], therefore showing that these proteins have an important mechanistic role in AD. All of these major enriched plaque proteins tightly correlate with Aβ levels in the brain [60] and SMOC1, OLFML3, NTN1 were recently identified as novel CSF biomarkers for AD [62, 128]. Together, these results show that these major enriched amyloid plaque proteins have excellent potential as novel drug targets and/or biomarkers for AD, and should be the focus of future studies.

One of these highly enriched plaque proteins—SMOC1—was the focus of our immunohistochemistry validation studies. The role of SMOC1 in AD and its function in the brain is currently unknown. Single cell RNAseq studies suggest that SMOC1 is enriched in oligodendrocyte precursor cells in the brain [130] and previous studies have highlighted its role in glucose homeostasis [131], angiogenesis [132] and ocular and limb development [133]. Here we show for the first time that it is highly enriched in a subpopulation of amyloid plaques. It is not yet known why SMOC1 co-localizes only with some plaques, but this could be due to SMOC1 interacting with a particular Aβ species such as pyroglutamate or phosphorylated Aβ. Indeed, our findings suggest that a large amount of SMOC1 immunoreactive amyloid plaques present in the hippocampus also contained post-translationally modified Aβ species. A hierarchical occurrence of Aβ1–40/42, pyroglutamate and pAβ throughout the course of AD has been proposed, suggesting that detection of pyroglutamate Aβ in amyloid plaques starts in preclinical AD, while phosphorylated Aβ preferentially starts aggregating in symptomatic AD [134]. Combined with our results, this might suggest that SMOC1 aggregation starts early in plaque development. A more comprehensive study looking at SMOC1 immunoreactivity in these plaque subtypes at different disease stages would provide a more definite answer. Together, our results provide further support for the important role of SMOC1 in AD and highlights the need for future studies to examine its mechanistic role in AD, particularly given the elevation of SMOC1 in the brain in preclinical AD [54]. Importantly, the finding that SMOC1 is not enriched in all plaques highlights the fact that not all amyloid plaques contain the same protein composition, which is consistent with our previous finding that plaques in rapidly progressive AD have a significantly different plaque protein expression than typical late onset AD [11].

We hypothesize that the proteins that are highly enriched in amyloid plaques have an important mechanistic role in AD pathology. A common criticism regarding the pathological importance of proteins that accumulate in plaques is that they may not have a mechanistic role in driving pathology and are simply present in plaques by chance. However, a comprehensive review of the literature does not support this criticism. 60% of the 48 proteins commonly enriched in plaques in EOAD and DS have already been confirmed to have a mechanistic role in driving AD pathology in transgenic mouse models or in vitro (Table 2). Previous studies show that 15 plaque enriched proteins pathologically promote Aβ aggregation/plaque formation or enhance Aβ associated toxicity. Notable examples include apolipoprotein E [82, 83], clusterin [7] and complement proteins (C1QB, C1QC, C3) [94, 99]. Conversely, previous studies show that 13 proteins are protective against AD pathology and can inhibit Aβ aggregation/plaque formation or protect against Aβ associated toxicity. For many of these proteins, previous research examining their mechanistic role in AD is limited to only a small number of studies. This suggests that plaque enriched proteins are not simply “tombstone markers” of disease, but instead can provide important insight into the factors that either drive or modulate the development of pathology in AD. Additionally, this also shows that proteins enriched in amyloid plaques are a mix of pathological and protective proteins and that enrichment in plaques does not automatically suggest a detrimental role in AD.

The core group of 48 proteins enriched in plaques in both DS and EOAD were highly enriched in extracellular proteins and endosomal-lysosomal system proteins. The enrichment of extracellular proteins is expected given the extracellular location of amyloid plaques. However, the significant enrichment of endosomal-lysosomal system proteins in plaques was intriguing. A growing body of evidence convincingly shows that Aβ accumulates intraneuronally within endolysosomal vesicles at early stages of AD [135, 136]. Endolysosomal vesicles provide an ideal environment for Aβ production and aggregation: it is the location where many of the key AD associated proteins colocalize (e.g. APP, presenilin-1), the acidic environment promotes Aβ aggregation and, the enclosed space promotes increased interaction and aggregation [137]. These observations have prompted the inside-out amyloid hypothesis, which proposes that the gradual accumulation of intraneuronal Aβ42 aggregates result in eventual synaptic/neuronal degeneration and the release of Aβ42 into the extracellular space which forms the nidus of amyloid plaques [135, 137–141]. Our finding of the enrichment of endolysomal proteins and other selected synaptic proteins in amyloid plaques in DS and EOAD supports this hypothesis.

Arl8b (encoded by the gene ARL8B) is an example novel lysosomal protein that we identified as enriched in amyloid plaques in both DS and EOAD. Arl8 is a small GTPase located on lysosomes that facilitates lysosomal trafficking along axons by acting as the linking molecule between lysosomes and kinesin-1 [142, 143]. Disruption of Arl8 function contributes to impaired lysosomal transport in axons, autophagic stress and neuron death in the neurodegenerative lysosomal storage disorder Niemann-Pick disease type C [144], confirming that it can contribute to neurodegenerative disease. The role of Arl8 in AD has not yet been studied and it has only been linked to AD in bulk-tissue ‘omics studies [54, 59]. Arl8a, the other paralog of arl8 in vertebrates, was also enriched in amyloid plaques in DS and showed a strong trend for enrichment in plaques in EOAD. Our finding that arl8b, as well as other endosomal-lysosomal proteins, were enriched in amyloid plaques provides additional support for the potential importance of lysosomes in the formation of amyloid plaques.

Our immunohistochemistry and literature validation studies showed that amyloid plaque enriched proteins had different colocalization patterns in amyloid plaques. For example, endolysosomal proteins typically have punctate/granular localization in plaques. This staining pattern was observed for ARL8B in our study, which was identical to the staining pattern seen for other lysosomal proteins in past studies such as cathepsin D [145], cathepsin B [146], LAMP1 [147], and lipofuscin [145], which is an accumulation of highly oxidized cross-linked molecules that accumulate in lysosomes during aging. The lack of colocalization of these lysosomal proteins with Aβ in plaques suggests that these lysosomal proteins may not be directly interacting  with Aβ, but may instead be located in small pockets in amyloid plaques where Aβ is either not present or in the process of being degraded. In contrast, SMOC1, moesin and ezrin showed high colocalization with Aβ fibrils in plaques, particularly in the plaque core, suggesting that these proteins likely interact directly with Aβ. A similar staining pattern was also observed in past studies for other major plaque proteins such as apolipoprotein E [81] and COL25A1 [148]. These results also highlight an important limitation of our study; designation as a “plaque-enriched protein” does not imply direct interaction with Aβ, instead this identifies a group of proteins that are significantly enriched in plaques in comparison to non-plaque tissue. While our immunohistochemistry validation results strongly suggest that some of these novel plaque-associated proteins interact with Aβ, future studies are required to confirm this.

Direct comparison of the amyloid plaque proteome in EOAD and DS showed that amyloid plaques in the two subtypes of younger onset AD had a very similar plaque protein composition. This shows that despite the different disease initiating factors, the resulting amyloid plaques still largely contain the same proteins. While some proteins were enriched to a much greater extent in amyloid plaques in either DS or EOAD (e.g. SMOC1), the trend for enrichment in both subtypes was highly similar. It is still unclear how these relative plaque protein differences influence AD pathogenesis. Future mechanistic studies examining how each of these proteins influence Aβ aggregation or clearance are needed. Future proteomic studies examining whether these major plaque enriched proteins are also enriched in other subtypes of AD (such as late onset AD, rapidly progressive AD or familial EOAD) would also potentially provide insight into differences into the rate, topography or type of plaque pathology between these subtypes.

In conclusion, we provide a new resource for the AD field that comprehensively characterizes proteins that are enriched in amyloid plaques in multiple subtypes of AD. We propose that these consistently enriched amyloid plaque proteins provide insight into the mechanisms driving amyloid plaque development in AD and are potentially novel drug targets and/or biomarkers for AD.

## Supplementary Information

**Additional file 1: Table S1**. Total imputed dataset. **Table S2**. Unimputed data. **Table S3**. Proteins enriched in plaques in both EOAD and DS (used to generate Figs. 2 and 4). **Table S4**. Proteins uniquely enriched in plaques in EOAD. **Table S5**. Proteins uniquely enriched in DS plaques. **Table S6**. Chromosome 21 proteins identified in our proteomic analysis. **Table S7**. DS vs EOAD plaque protein differences.

**Additional file 2: Figure S1**. *APOE* genotyping. (A) Schematic diagram of the *APOE* genotyping methodology. Six scrolls of 8 µm were sectioned from FFPE blocks and DNA was isolated with the automated QIAsymphony SP. An endpoint PCR was performed, samples were resolved in a 2% agarose gel and amplified DNA was purified from the gel. A second PCR with the purified DNA was performed and un-purified PCR products were sequenced to determine the *APOE* genotype. (B) Representative gel of EOAD and DS samples used for sequencing. *APOE* band is located at 348 bp. (C) Sanger sequencing chromatogram showing the nucleotides located in the single-nucleotide polymorphisms (SNPs) rs429358 and rs7412, which determine the *APOE* variant *ε*3.
