# Supplementary figures and images for: The amyloid plaque proteome in early onset Alzheimer’s disease and Down syndrome
Source: Acta Neuropathol Commun. 2022 Apr 13;10:53. doi: 10.1186/s40478-022-01356-1 (PMC9008934; doi:10.1186/s40478-022-01356-1)

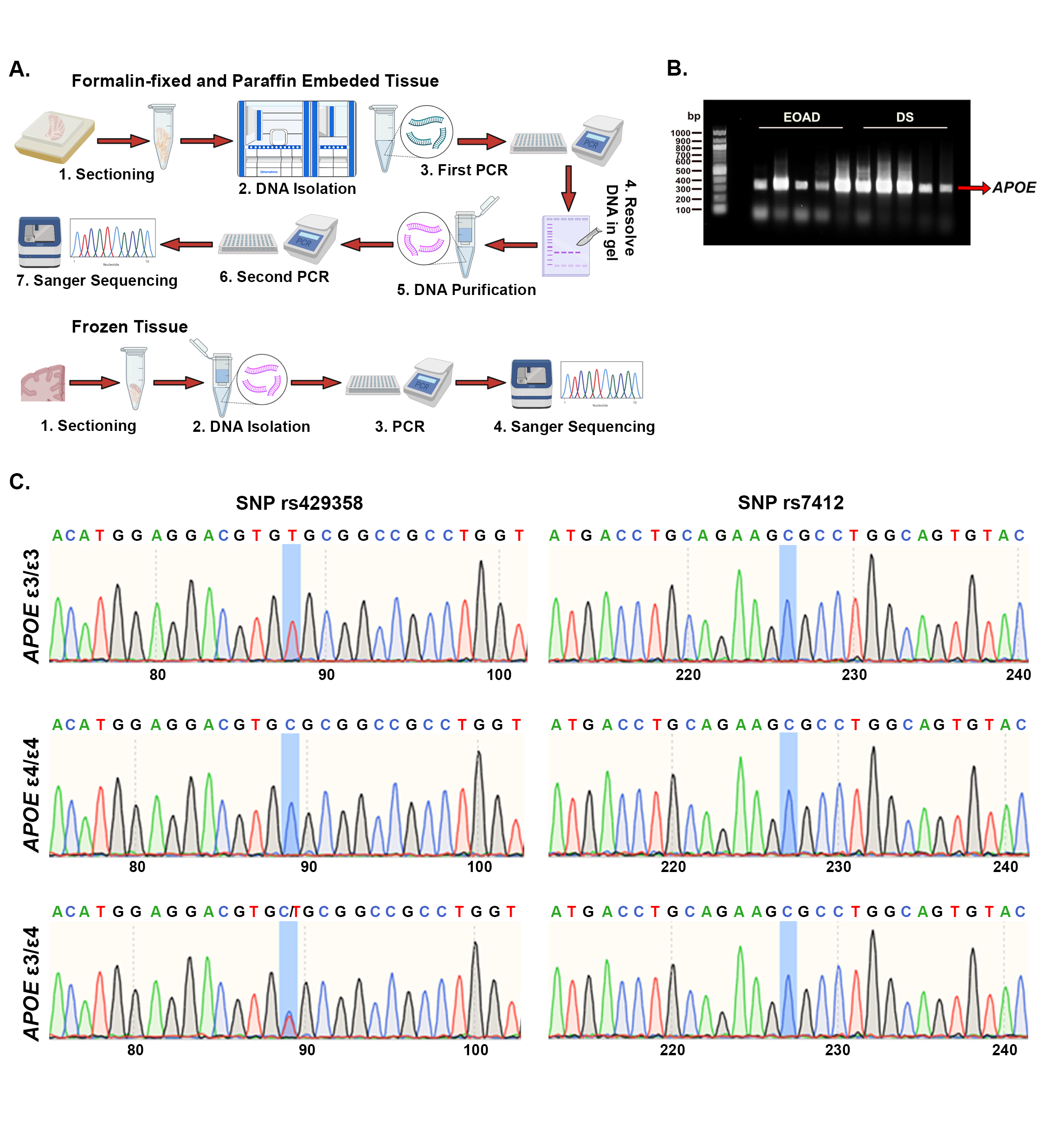

Supplement: Supplementary file 2 — Additional file 2: Figure S1. APOE genotyping. (A) Schematic diagram of the APOE genotyping methodology. Six scrolls of 8 µm were sectioned from FFPE blocks and DNA was isolated with the automated QIAsymphony SP. An endpoint PCR was performed, samples were resolved in a 2% agarose gel and amplified DNA was purified from the gel. A second PCR with the purified DNA was performed and un-purified PCR products were sequenced to determine the APOE genotype. (B) Representative gel of EOAD and DS samples used for sequencing. APOE band is located at 348 bp. (C) Sanger sequencing chromatogram showing the nucleotides located in the single-nucleotide polymorphisms (SNPs) rs429358 and rs7412, which determine the APOE variant ε3. [file 40478_2022_1356_MOESM2_ESM.tif]
